# Edge‐Grafted Polyarginine Functionalization of Graphene Nanocarriers Maintains Noncovalent Aromatic Drug Loading

**DOI:** 10.1002/psc.70114

**Published:** 2026-07-05

**Authors:** Beatrice Scagnoli, Alessandro Semeraro, Kaiyue Hu, Alberto Ongaro, Agnese Pavan, Luigi Brambilla, Chiara Castiglioni, Maria Cristina De Rosa, Giuseppe Pappalardo, Giuseppina Sabatino, Michele Maggini

**Affiliations:** ^1^ Istituto di Scienze e Tecnologie Chimiche Giulio Natta (SCITEC)‐CNR Rome Roma Italy; ^2^ Dipartimento di Chimica, Materiali e Ingegneria Chimica Giulio Natta Politecnico di Milano Milan Italy; ^3^ Dipartimento di Scienze Chimiche Università di Padova Padova Italy; ^4^ CNR‐Istituto di Cristallografia Catania Italy; ^5^ Istituto di Chimica della Materia Condensata e di Tecnologie per l'Energia (ICMATE)‐CNR Padova Italy

**Keywords:** cell penetrating peptides, drug delivery, FT‐IR spectroscopy, graphene nanoparticles, molecular dynamics simulations, NEK6 inhibitors, UV–vis spectroscopy

## Abstract

Covalent peptide grafting is widely used to improve the biological performance of nanocarriers, especially through cell‐penetrating peptides (CPPs) that enhance cellular uptake. However, when drug loading relies on noncovalent interactions, peptide functionalization may interfere with surface adsorption processes. This concern is particularly relevant for graphene‐based nanocarriers, where π‐conjugated molecules bind via π–π interactions on the basal plane. Here, we present an orthogonal functionalization strategy in which peptide conjugation occurs selectively at the edges of graphene nanoparticles (B60), which bear carboxylic acid groups, while π‐conjugated cargo is adsorbed on the basal plane. Poly‐arginine‐11 (R11) was covalently immobilized, preserving the aromatic surface for π‐π interactions. Using 1‐pyrenecarboxylic acid and compound **8**, a π‐conjugated NEK6 inhibitor, we show that R11 grafting does not affect loading capacity or thermally induced release. Spectroscopic and microscopic analyses confirm that the basal plane remains intact and accessible after functionalization. Molecular dynamics simulations indicate that peptide chains form a flexible, charged corona at the nanoparticle periphery without perturbing molecule–graphene interactions. Overall, edge‐grafted R11 preserves π‐π‐mediated loading and supports the design of peptide–graphene hybrid systems for delivering poorly soluble aromatic bioactive compounds.

## Graphical abstract

Edge‐selective covalent grafting of poly‐arginine‐11 onto B60 graphene nanoparticles preserves the aromatic basal plane for π‐π immobilization of compound **8**, highlighting this promising NEK6 inhibitor as the key therapeutic cargo in a peptide‐graphene nanoplatform for potential delivery.

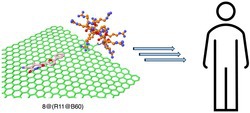

## 1 Introduction

**Chart 1:**
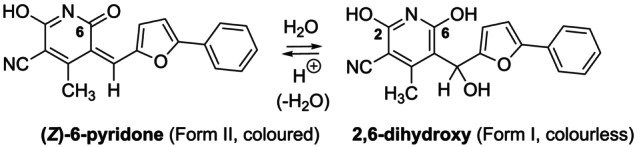
Molecular structures of NEK6 inhibitors **8**. The numbers are taken from reference [19].

Integration of peptides with nanomaterials enables the design of hybrid systems that couple tuneable biological specificity with defined physicochemical properties [1, 2]. Peptides in nanomedicine can be exploited in two principal ways: as building blocks that self‐assemble into nanostructured materials [3, 4], or as functional moieties to modify preformed nanomaterials, thereby imparting targeting capabilities, antimicrobial properties, and improving cellular uptake [5]. Cell‐penetrating peptides (CPPs) are particularly valuable in this context as short sequences able to cross biological barriers and facilitate intracellular delivery of molecular cargo. Among CPPs, oligoarginine sequences exhibit an especially high internalization efficiency compared with other cationic peptides based on lysine, ornithine, or histidine [6]. This behavior is strongly dependent on chain length, with uptake increasing to an optimal range of approximately 11 residues, making poly‐arginine‐11 (R11) one of the most effective scaffolds [7]. Internalization of R11 is mainly driven by electrostatic interactions between guanidinium groups and negatively charged membrane components. Additional mechanisms, including membrane‐assisted translocation pathways and interactions with membrane proteins, have also been proposed [8]. In this context, graphene‐based nanomaterials (GBNs), including graphene oxide and related derivatives, have attracted increasing interest in biomedicine due to the structural features that make them suitable platforms for molecular transport [9, 10]. Their large surface area and extended aromatic domains enable efficient loading of aromatic/π‐conjugated therapeutic agents through π‐π interactions [11]. In addition, oxidized graphene derivatives expose oxygen‐containing groups, mainly located at sheet edges and defect sites, which allow for covalent functionalization, while the remaining aromatic basal plane remains available for noncovalent adsorption. This dual chemical character provides a versatile framework for the design of graphene‐based drug delivery systems [12, 13]. R11 is particularly well suited for integration with GBNs because its guanidinium‐rich cationic structure complements the chemistry of graphene edges without requiring modification of the aromatic basal plane. This combination is particularly attractive because it couples two complementary functions: the surface of graphene can act as a loading platform for aromatic/π‐conjugated molecules, while the peptide component can improve the interaction with biological membranes and promote uptake [14, 15]. This structural arrangement enables peptide grafting at the edges of the sheet while preserving the aromatic basal plane for π‐π adsorption of payload molecules. For this reason, the potential interference of edge‐bound peptides with basal plane drug adsorption must be carefully evaluated rather than assumed. This point is critical because most of the drug delivery systems based on graphene reported so far rely on graphene oxide or related oxidized derivatives, whose heterogeneous structure and surface chemistry are often difficult to control and result in limited reproducibility [16]. To the best of our knowledge, structurally defined graphene nanoparticles with an intact aromatic basal plane have rarely been explored as drug delivery platforms. To address this limitation, we recently developed a simple, cost‐effective, and environmentally friendly method for the preparation of graphene‐based nanoparticles (hereafter referred to as B60), characterized by an average lateral size of ~120 nm and about six graphene layers. These nanoparticles spontaneously generate peripheral carboxylic acid groups that enable efficient covalent conjugation of R11. At the same time, the graphene basal plane remains intact, establishing an orthogonal functionalization framework [17, 18]. In this architecture (hereafter referred to as R11@B60), peptide conjugation occurs at the particle edges, while the basal plane remains available for π‐π‐driven immobilization of aromatic/π‐conjugated small molecules, allowing independent tuning of biological interactions and cargo adsorption. Once such a platform has been established, the next question is which biologically relevant cargo molecules can benefit the most. In this regard, inhibitors of Never In Mitosis A‐related kinase 6 (NEK6) represent especially interesting candidates. NEK6 is a serine/threonine protein kinase involved in spindle assembly and chromosomal stability and implicated in cancer progression and drug resistance. Among the 25 potential hit compounds identified through a computer‐aided drug design campaign targeting NEK6 [19], compound **8** (Chart 1) emerged as a particularly promising lead. Beyond its reported antiproliferative and chemosensitizing activities [19], subsequent studies further highlighted its therapeutic potential in cardiac dysfunction [20] and amyotrophic lateral sclerosis (ALS) [21].

However, compound **8** exhibits poor aqueous solubility, which limits its formulation, handling, and biological application. Therefore, immobilization within a suitable nanocarrier may represent an effective strategy to enhance its pharmacological potential. In this context, efficient delivery systems for NEK6 inhibitors are relevant across multiple disease settings. In the absence of any peptide functionalization, we demonstrated that compound **8** can be efficiently immobilized on bare B60 through π–π interactions, as non‐covalent complex named **8**@B60 [22]. Based on this result, the introduction of R11 adds a complementary functionality. The extended aromatic surface of graphene interacts with the π‐conjugated inhibitor **8** and may improve its dispersion. At the same time, the R11 component may enhance cellular interaction and uptake. However, this combined architecture also raises a nontrivial question: peptide decoration, while beneficial for biological transport, could in principle interfere with drug loading by introducing competing peptide–drug interactions or by altering the colloidal behavior of the graphene dispersion. Based on these considerations, the present study investigates R11‐decorated B60 as a carrier for NEK6 inhibitors. Specifically, we examine the formation of the non‐covalent 8@(R11@B60) system, in which compound **8** is adsorbed onto the graphene platform. A model system based on 1‐pyrenecarboxylic acid (PyCA) was also used to probe π–π interactions in a simplified context, leading to the formation of the PyCA@(R11@B60) complex. For clarity, the notation used throughout this work does not by itself specify the nature of the interactions among the components. R11@B60 denotes the covalently functionalized graphene platform obtained by grafting R11 to the edge carboxyl groups of B60. In contrast, **8**@(R11@B60) and PyCA@(R11@B60) denote assemblies in which compound **8** or PyCA are non‐covalently adsorbed onto the graphene surface of the R11‐functionalized platform. Our aim is to assess whether the graphene platform, featuring covalent edge functionalization with R11 and noncovalent adsorption in the basal plane, can support efficient loading and controlled release of these poorly water‐soluble NEK6 ligands. The interpretation of the experimental results was further supported by molecular modeling and molecular dynamics (MD) studies, providing structural insight into the spatial organization of the peptide corona and its influence on drug adsorption at the graphene basal plane.

## 2 Materials and Methods

PyCA and all other reagents were purchased from Merck and used as received.

### 2.1 Preparation of B60 and 8@B60

B60 [17] and the π‐π system **8**@B60 [22] were prepared according to previously reported procedures.

### 2.2 Preparation of R11@B60

The covalently functionalized B60 system was prepared by the method reported previously [18]. Briefly, R11 was synthesized by solid‐phase peptide synthesis, following the fluorenylmethoxycarbonyl (Fmoc)/*tert*butyl strategy, on 0.1 mmol of Rink amide AM resin (0.35 mmol/g) and using a fully automated microwave‐assisted peptide synthesizer (Liberty Blue 2.0, CEM Corporation, Matthews, NC, United States). R11 was covalently grafted onto B60 through EDC/NHS‐mediated amidation between the native carboxyl groups at the edges of the graphene layers and the N‐terminal amino group of the peptide.

### 2.3 Preparation of 8@(R11@B60)

R11@B60 powder (3.1 mg) and compound **8** (0.62 mg) were dispersed in CH_3_OH (2 mL) and sonicated in an ultrasonic bath for 45 min. Sterile water (6.2 mL) was then added, and sonication was continued for 24 h. The resulting aqueous dispersion of **8**@(R11@B60) was washed repeatedly to remove excess unbound **8** and residual CH_3_OH. The washing procedure, described in detail in Ref. [18], was performed for *n* cycles (*n* = 1–4), affording the final dispersions denoted as **8**@(R11@B60_nW).

The loading efficiency was estimated using two independent methods. In the first approach, the amount of free compound **8** remaining in the aqueous dispersion after preparation of **8**@(R11@B60), but prior to the washing steps, was quantified, yielding a loading efficiency of η = 92%. In the second approach, the concentration of compound **8** released into the aqueous phase after thermal treatment of **8**@(R11@B60) and subsequent nanoparticle removal by centrifugation was measured, providing a lower estimate of η = 46%. Details of both procedures are reported in the Supporting Information. The two values reflect the different assumptions underlying the corresponding experimental approaches. The first estimate is likely to overestimate the loading efficiency because it does not account for the loss of weakly bound molecules during the washing steps. Conversely, the second estimate may underestimate the loading efficiency because complete thermal release of compound **8** from the graphene platform cannot be guaranteed. Therefore, the actual loading efficiency is expected to lie between these two estimates.

### 2.4 Preparation of PyCA@(R11@B60)

The model system PyCA@(R11@B60), in which PyCA is adsorbed on the graphene platform, was obtained using the same protocol described above. R11@B60 powder (4 mg), 1‐pyrenecarboxylic acid (0.66 mg), and sterile water (8 mL) were used. The resulting dispersion was subjected to n washing cycles (*n* = 1–4) to remove excess unbound PyCA and residual CH_3_OH, yielding the final dispersions denoted as PyCA@(R11@B60_nW).

### 2.5 Morphology of 8@(R11@B60)

The morphology and microstructure of **8**@(R11@B60) was investigated by transmission electron microscopy (TEM), using a JEOL F200 microscope operated at 200 kV. Samples were prepared by depositing an aqueous suspension of the system onto a 400‐mesh lacey carbon grid.

### 2.6 Spectroscopic Characterization

Aqueous dispersions of **8**@(R11@B60) and PyCA@(R11@B60) were characterized by UV–vis absorption spectroscopy using a JASCO V‐570 spectrophotometer (Japan). Measurements were performed in 3.5 mL quartz cuvettes with an optical path length of 1 cm. IR spectra were collected in specular reflection (SR) mode using a Thermo Nicolet 6700 FT‐IR spectrometer coupled to a ThermoElectron‐Nicolet Continuμm FT‐IR microscope (15× infinity‐corrected Cassegrain objective, 512 scans, spectral resolution 4 cm^−1^). SR measurements were performed on homogeneous flat films obtained by drying a few drops of the aqueous dispersions. To ensure collection of purely specularly reflected IR radiation, spectra were recorded on films with thicknesses of several hundred micrometers. Specular reflectivity spectra were converted into absorbance by applying the Kramers–Kronig (KK) transformation using the OMNIC 8.0 software. IR spectra of solid **8** were registered with the same instrumentation using a diamond anvil cell (DAC) accessory. CHCl_3_ solutions were analyzed in transmission mode using a 0.1 mm KBr‐window liquid IR cell.

### 2.7 Thermal Release Experiments

Thermal release of PyCA@(R11@B60) was investigated by heating an aqueous dispersion at 50°C for 60 min. The sample was prepared as described above and subjected to four washing cycles. Release of PyCA from the graphene platform was confirmed by the increase in the fluorescence signal of free PyCA in water. Thermal release of **8**@(R11@B60) was investigated by heating an aqueous dispersion at 40°C for 90 min. The sample was prepared as described above and subjected to four washing cycles, without significant loss of the payload. Release of compound **8** was monitored through changes in the UV–Vis absorption profile.

### 2.8 Simulation Methods

#### 2.8.1 System Set‐Up

Graphene sheets with square dimensions of 50 × 50 Å were generated using the Python script Make‐Graphitics, available on GitHub (https://github.com/velocirobbie/make‐graphitics, [23]). As part of the model‐building procedure, the generated structures were also preliminarily energy‐minimized with the OPLS force field [24] yielding well‐defined models for the subsequent computational analyses. To investigate the effect of R11 on the loading of **8**, a second graphene‐based system was developed. In this system, a carboxylic acid group was first introduced at one edge of the graphene sheet to create a suitable reactive site for further derivatization. The graphene was then functionalized through the covalent attachment of R11, resulting in a hybrid construct in which the graphene scaffold was associated with a cationic peptide moiety [18]. The structure of R11, as well as the associated molecular modeling workflow, was generated and processed using BIOVIA Discovery Studio 2025 (BIOVIA, Dassault Systèmes, Discovery Studio, Discovery Studio 2025, San Diego: Dassault Systèmes, 2025), which enabled the preparation of the graphene models. Compound **8** was prepared using the Prepare Ligands protocol implemented in BIOVIA Discovery Studio 2025 under physiological conditions (pH 7.0), thereby ensuring the assignment of the correct protonation state and the generation of an optimized geometry prior to its incorporation into the system. Following molecular modeling, **8** was incorporated into the system in the conformation previously identified as the most stable for interaction with graphene [22]. To generate the **8**@(R11@B60) system, compound **8** was positioned on the graphene surface of the R11‐functionalized construct using this adsorption geometry. The initial configuration therefore consisted of compound **8** adsorbed onto the graphene platform, while the covalently attached R11 chain remained free to explore the surrounding solvent environment.

#### 2.8.2 MD

In each system, the MD simulations were performed by the Desmond software package (Schrödinger Release 2025‐1: Desmond Molecular Dynamics System, D. E. Shaw Research, New York, NY, 2025) as implemented in Maestro. The systems were embedded in cubic simulation boxes with a minimum 10 Å buffer between the solute and the box edges, solvated with the TIP3P water model, and parameterized using the OPLS4 force field. Electrical neutrality was ensured by the addition of Cl^−^ counterions, and physiological salt conditions (0.15 M NaCl) were reproduced by introducing the appropriate numbers of Na^+^ and Cl^−^ ions. The time step of each MD simulation was 2 fs, and periodic boundary conditions (PBC) applied in all three dimensions. Long‐range electrostatic interactions were treated using the Particle Mesh Ewald (PME) method [25] and a cutoff radius of 9.0 Å was applied for short‐range van der Waals and Coulomb interactions. Each system was first energy‐minimized and subsequently equilibrated using the default Desmond relaxation protocol. Production MD simulations were then carried out under NPT conditions for 300 ns, with all simulations performed in triplicate to ensure the reproducibility of the results. The temperature (300 K) and pressure (1 atm) were controlled using the Nosé‐Hoover chain thermostat and the Martyna‐Tobias‐Klein barostat, respectively. The simulation trajectories were analyzed and the corresponding time‐evolution profiles were generated using the Simulation Interaction Diagram tool available in Maestro Schrödinger, VMD [26] for data extraction, and Python for subsequent data processing and plotting. Trajectory clustering analysis was performed using the Desmond Trajectory Clustering tool implemented in Schrödinger Maestro. Frames were grouped based on pairwise RMSD values calculated for the R11 chain, considering every fifth frame of the trajectory. Clustering of the resulting RMSD matrix was then carried out to identify representative conformational states sampled during the simulation.

## 3 Results and Discussion

**FIGURE 1:**
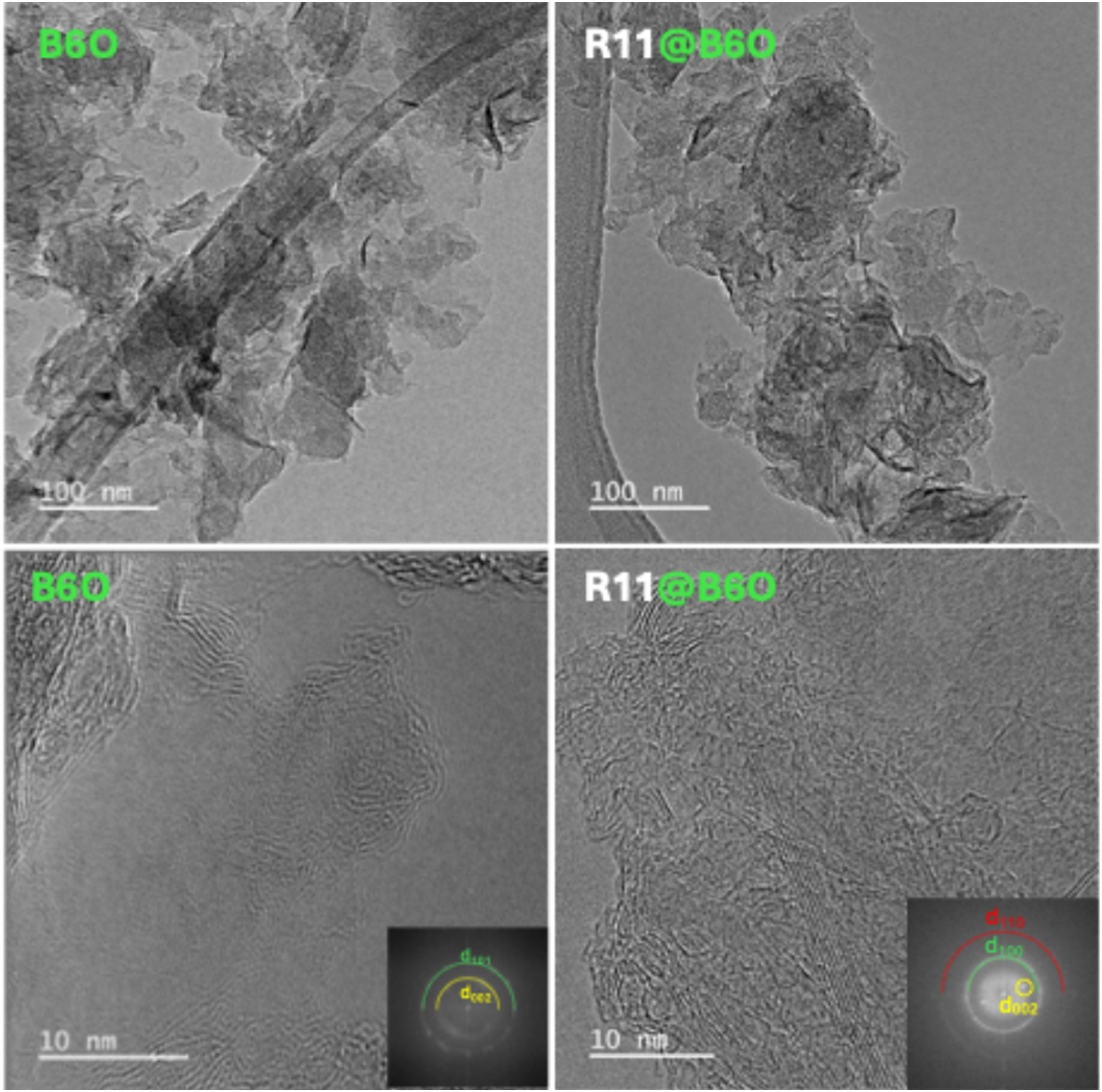
TEM images of B60 and R11@B60 at low and high magnification. The morphology of the nanoparticles is preserved after functionalization of B60 with R11. High‐magnification images reveal short‐range lattice fringes, indicative of residual graphitic order originating from ball‐milled graphite. The corresponding diffraction patterns (insets) show reflections assigned to the (002), (101) and (110) planes, confirming a multilayer structure and in‐plane periodicity.

The PyCA@(R11@B60) and **8**@(R11@B60) systems were investigated to characterize the loading behavior of aromatic guest molecules on the R11‐functionalized B60 platform. Both PyCA and compound **8** are π‐conjugated molecular structures that enable π–π interactions with the graphene domains of B60, a feature previously shown to drive the adsorption of these molecules onto bare B60 [18, 22]. These earlier studies provide a reference framework for assessing the behavior of the corresponding systems after functionalization with R11 of B60. The inclusion of R11 was motivated by the need to promote efficient intracellular delivery of compound **8**. R11 was selected because of its well‐documented ability to facilitate translocation across the plasma membrane in a variety of cell types. Among cationic CPPs, oligoarginines exhibit particularly high uptake efficiency, with R11 representing one of the most effective variants [6]. This behavior is strongly dependent on chain length, and the 11‐residue sequence has been identified as an optimal scaffold for intracellular transport [7]. A key structural aspect of the system is that R11 functionalization occurs at the edges of the graphene layers, through covalent coupling with native carboxylic groups present at the nanoparticle periphery. As a result, the basal plane of the graphene sheets should remain, at least in principle, preserved and available for π–π interactions with aromatic guest molecules. This spatial separation between edge functionalization and basal‐plane adsorption is expected to allow peptide grafting without significantly perturbing the loading properties of the graphene platform. This structural picture will be further rationalized by molecular modeling results discussed later in this section. Covalent functionalization of B60 with R11 was accomplished through EDC/NHS‐mediated coupling chemistry, which provides high conjugation efficiency under mild aqueous conditions and yields stable amide bonds. The structural features of R11@B60 have been established in previous work [18]. Spectroscopic characterization by UV–vis, IR and Raman spectroscopy confirmed the successful covalent grafting of the peptide onto B60. Raman analysis demonstrated that the graphene layers remain structurally intact after functionalization, while TEM revealed that the morphology of R11@B60 closely resembles that of pristine B60 (Figure 1).

Loading of π‐conjugated **8** and PyCA onto R11@B60 was examined using complementary spectroscopic techniques. UV–vis absorption spectroscopy was employed to analyze aqueous dispersions of the systems. The spectra display the characteristic absorption features of both R11@B60 and the loaded molecular species. Importantly, the absorption bands associated with the guest molecules remain detectable even after repeated washing cycles, indicating the presence of stable interactions between the aromatic guests and the R11@B60 platform. In the case of compound **8**, the UV–vis spectra provide additional insight. Previous studies showed that this molecule can exist in a tautomeric, orange‐red form (Chart 1), which is normally unstable in aqueous media and rapidly converts to a colorless, hydrated form [22]. In the dispersions of the assemblies, however, the absorption features associated with the colored species remain clearly observable, indicating that interaction with the graphene surface stabilizes this π‐conjugated form. The spectroscopic properties and interconversion behavior of these forms have been discussed in detail in our previous work [22]. Further information on the nature of the assemblies was obtained by IR spectroscopy performed on solid samples prepared by deposition of the dispersions. This technique allows identification of the characteristic vibrational fingerprints of the loaded molecules. In some cases, the spectral features suggest that the guest molecules are present as isolated species rather than aggregated domains, indicating that strong molecule–platform interactions can inhibit self‐aggregation of the aromatic compounds. Release behavior was investigated by monitoring aqueous dispersions of the systems by UV–vis spectroscopy during controlled heating. In the case of PyCA, release from the graphene surface is accompanied by a marked increase in fluorescence intensity, consistent with the recovery of the emissive properties of the molecule upon detachment from the platform. For the conjugate **8**@(R11@B60), a dedicated protocol was developed to determine the uptake‐release yield of compound **8**, as described in the Supplementary Information.

### 3.1 The Model Assembly PyCA@(R11@B60)

**FIGURE 2:**
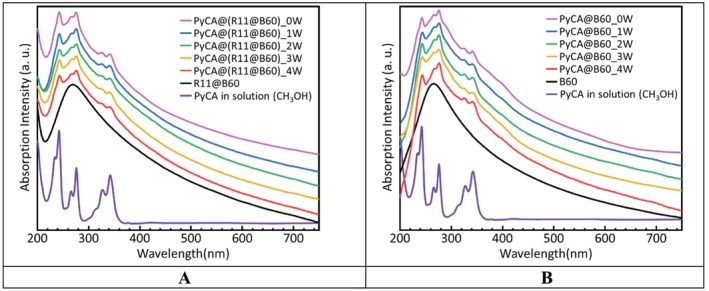
UV–vis spectra of aqueous dispersions of (A) PyCA@(R11@B60)_nW and (B) PyCA@B60_nW systems recorded after successive washing cycles (nW, *n* = 0–4). The spectra result from the superposition of the absorption bands of the graphene platform (R11@B60 or B60) and those of PyCA, whose spectrum is also shown for comparison. A moderate decrease in the intensity of the PyCA absorption bands is observed after the first washing step, whereas subsequent washing cycles produce nearly unchanged spectral profiles.

The UV–vis spectra of PyCA@(R11@B60) closely resemble those of PyCA@B60), the assembly formed with non‐functionalized B60 (Figure 2). In both cases, the spectra clearly display the characteristic absorption features of PyCA superimposed on the typical B60 absorption band, confirming the successful formation of the supramolecular assemblies. In the spectrum of PyCA@(R11@B60), the presence of the peptide is evidenced by the steep increase in absorption near 200 nm, corresponding to the low‐wavelength edge of the UV absorption of R11. To verify that the PyCA signals arise from molecules loaded onto the platform rather than from free species in solution, the systems were subjected to multiple washing cycles. A similar behavior is observed for both PyCA@(R11@B60) and PyCA@B60: a moderate decrease in the intensity of the PyCA absorption bands is detected when comparing the 0 W sample (before washing) with the nW samples (after *n* washing cycles). However, the spectral profile remains essentially unchanged from the second washing step onward. This behavior shows that free PyCA molecules in solution are largely removed during the first washing step, whereas PyCA molecules bound to the graphene surface remain associated with the platform and are not detached by subsequent washing.

**FIGURE 3:**
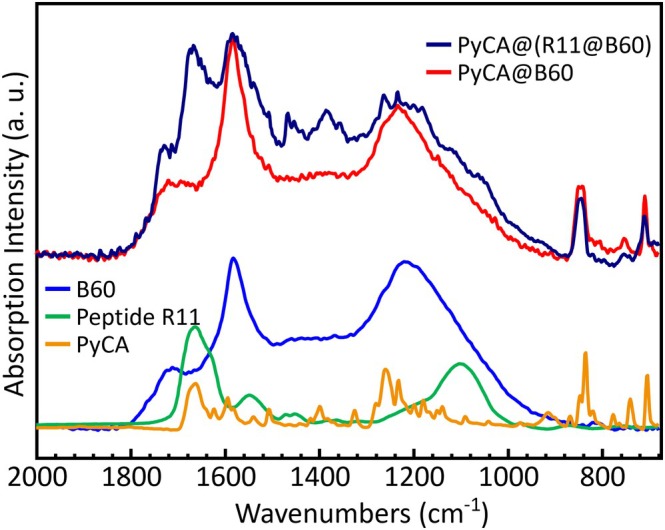
IR spectra of solid samples of PyCA@(R11@B60) (dark blue line) and PyCA@B60 (red line). For comparison, the spectra of the individual components—PyCA (orange line), R11 (green line), and bare B60 (light blue line)—are also shown, stacked in the lower part of the figure.

IR spectroscopy provides further evidence for the formation of the assemblies (Figure 3). Comparison of the spectra of PyCA@(R11@B60) and PyCA@B60 with those of the individual components (B60, R11, and PyCA) shows that the characteristic absorption bands of PyCA are clearly recognizable in both systems. At the same time, subtle changes in the IR pattern of loaded PyCA relative to the spectrum of the pure PyCA powder indicate that the molecule is adsorbed on the platform as isolated species rather than in the crystalline state. In the spectrum of PyCA@(R11@B60), additional features attributable to the R11 peptide are also observed, further confirming the successful covalent grafting of the peptide onto the graphene nanoparticles.

Complementary fluorescence experiments were used to monitor the thermal release of PyCA (see Supporting Information, Figure S1). In line with previous observations reported in Ref. [18], heating the PyCA@(R11@B60) dispersion at 50°C for 60 min leads to a pronounced increase in fluorescence intensity. This behavior confirms both the efficient loading of PyCA onto the R11@B60 platform, where fluorescence is quenched due to π–π interactions with the graphene surface, and its thermally triggered release, which restores the strong emission of the free molecule once detached from the platform. Moreover, the effectiveness of heating in promoting PyCA release provides further evidence that π–π interactions are primarily responsible for the loading of PyCA onto the platform. This behavior is inconsistent with the formation of stable covalent adducts arising from condensation reactions involving the arginine residues of R11, supporting a predominantly supramolecular mode of association.

### 3.2 The Drug‐Loaded System 8@(R11@B60)

**SCHEME 1:**
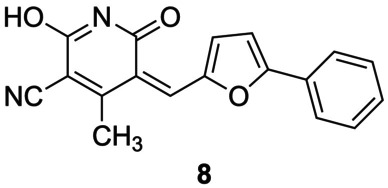
Reversible hydration of compound **8** upon acid addition.

Compound **8** exists as multiple conformational isomers and tautomers, and its spectroscopic identification required a dedicated analysis of the response of the most relevant forms [22]. The compound is readily soluble in methanol but only sparingly soluble in water. In aqueous media, it undergoes a reversible transformation between an orange‐colored form (Form II), characterized by an absorption band at 480 nm, and a hydrated colorless form (Form I) with a maximum at 330 nm (Scheme 1). Previous studies showed that adsorption onto B60 stabilizes the colored form of compound **8** [22].

As a result, the UV–vis spectrum of **8**@B60 shows a broadened absorption band that appears around 500 nm on the tail of the characteristic B60 band (λ_max_ = 265 nm), extending toward the near‐IR region. The persistence of this feature over time indicates that the **8**@B60 system forms stable aqueous dispersions at room temperature.

**FIGURE 4:**
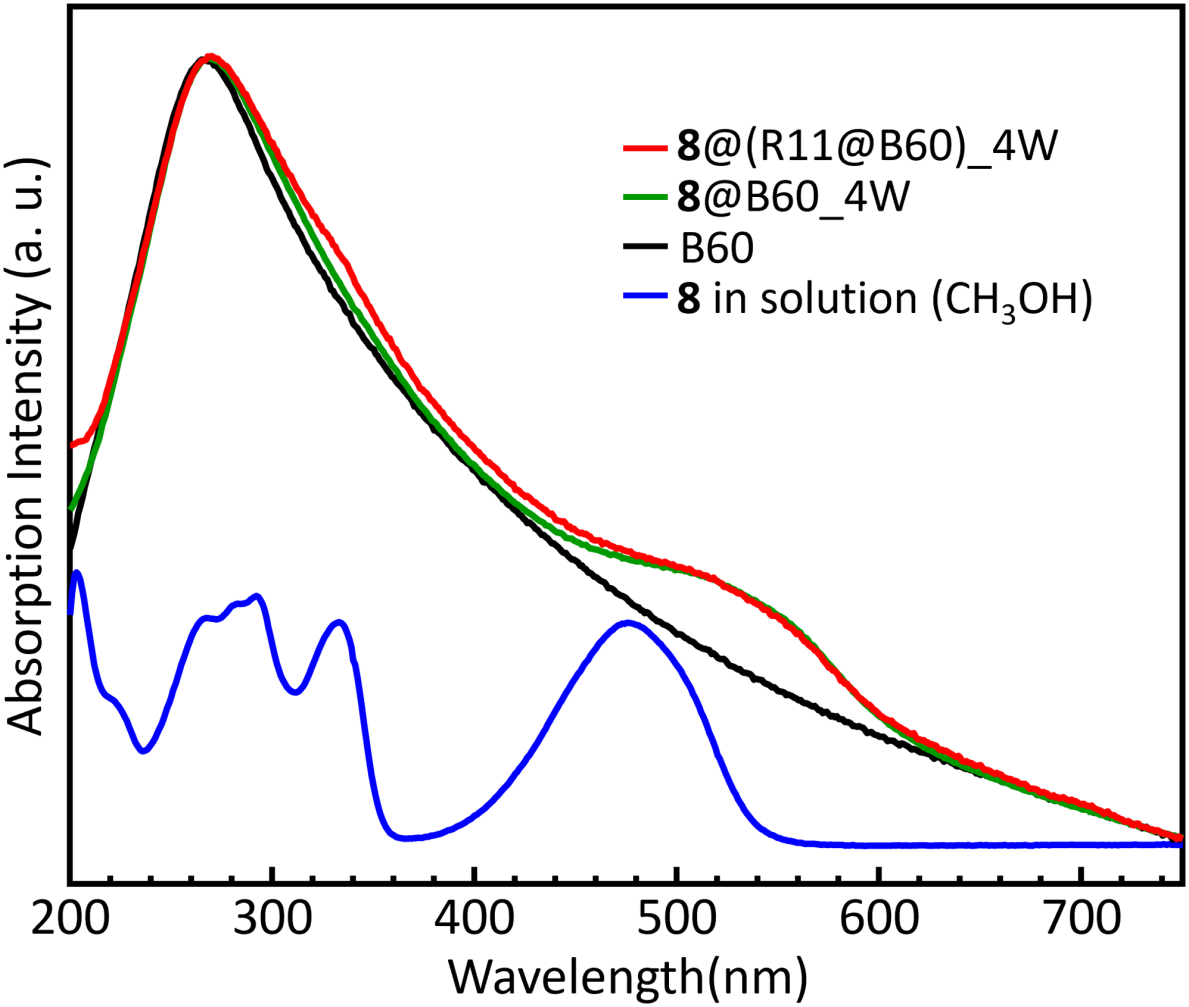
UV–vis spectrum of an aqueous dispersion of **8**@(R11@B60) after four washing cycles (red line). The spectral profile result from the superposition of the absorption bands of B60 and those of free compound **8**, which are also shown for comparison (black and blue lines, respectively). The UV–vis spectrum of **8**@B60 (green line) is additionally included for comparison.

UV–vis analysis of **8**@(R11@B60) dispersions reveals a spectral behavior closely resembling that observed for **8**@B60 (Figure 4). In particular, the spectral profile remains essentially unchanged after the second washing cycle, indicating that successive washing steps remove unbound molecules and that the dispersion contains only compound **8** associated with the platform (Figure S2). The loading of compound **8** onto R11@B60 was estimated by UV–vis spectroscopy using two complementary approaches based on characteristic absorption bands of **8**. The analyses indicate efficient conjugation, with loading yields in the range of ~46%–92% depending on the method (see Supporting Information, Figures S3–S4).

**FIGURE 5:**
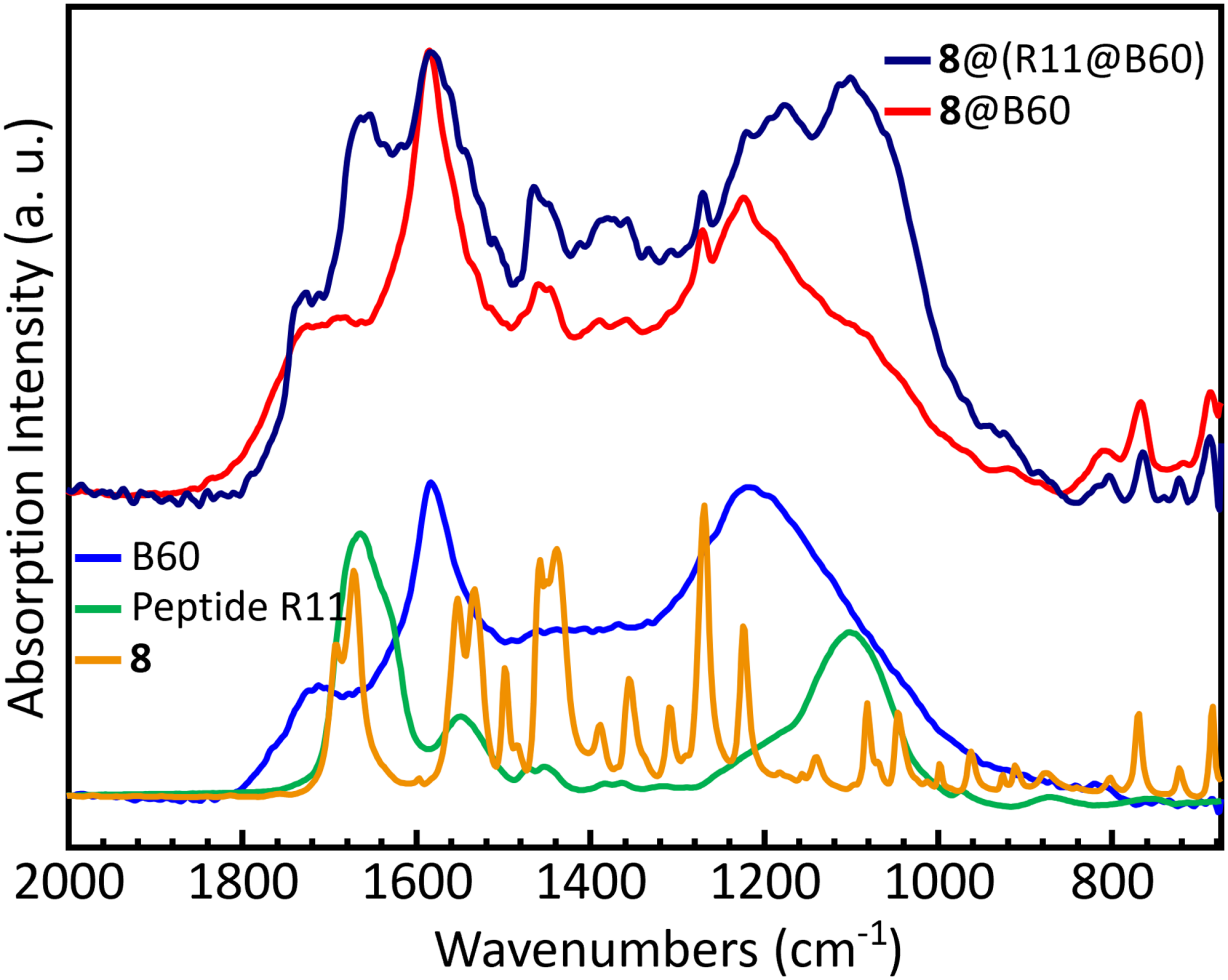
IR spectra of solid samples of **8**@(R11@B60) (dark blue line) and **8**@B60 (red line). For comparison, the spectra of the individual components—compound **8** (orange line), R11 (green line), and bare B60 (light blue line)—are also shown, stacked in the lower part of the figure.

The IR spectrum of **8**@(R11@B60) clearly displays the characteristic features of compound **8** adsorbed on the platform (Figure 5). Taken together, the results shown in Figures 4 and 5 indicate that, even for molecules with a more complex structure than the model compound PyCA, featuring greater conformational flexibility and multiple polar groups, the dominant interaction with the graphene surface remains π‐type, despite the presence of peptide residues that could potentially establish competing interactions with **8**. Further insight into these interactions will be provided by MD simulations on selected model structures, discussed at the end of this section.

**FIGURE 6:**
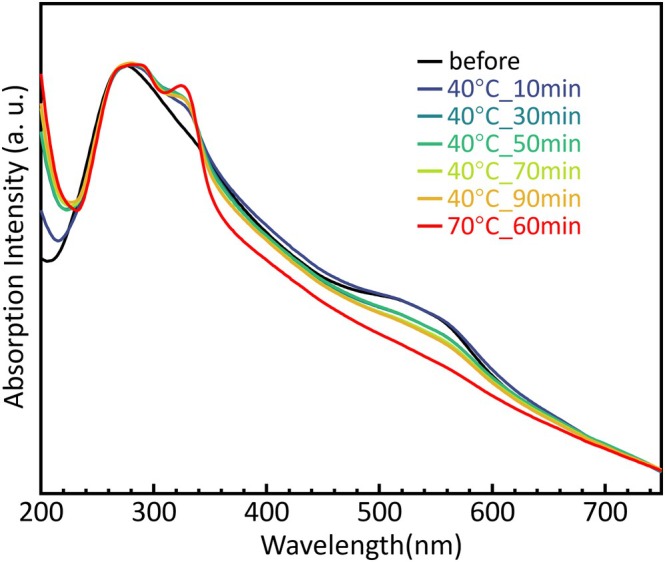
Evolution of the UV–vis spectra of an aqueous dispersion of **8**@(R11@B60) during heating at 40°C for 90 min. Spectra were recorded at 10 and 20 min intervals. The spectrum obtained after heating at 70°C for 60 min (red line) is also shown for comparison.

The release of compound **8** from **8**@(R11@B60) was evaluated by monitoring the UV–vis spectrum of the aqueous dispersion upon heating. As shown in Figure 6, the characteristic absorption of the Form II of **8** (orange‐colored) at ~500 nm progressively decreases with time while the sample is maintained at 40°C in a thermostatic bath. This broad feature completely disappears when the temperature is increased to 70°C. At the same time, a well‐defined absorption band at 330 nm emerges, indicating that the released molecules of **8** undergo the expected transformation in water to the colorless hydrated form. Comparison with our previous study on **8**@B60 [22] indicates that the presence of R11 grafted onto the graphene platform does not interfere with the thermally induced release of compound **8**.

### 3.3 Dynamics of 8@B60) and 8@(R11@B60) Systems

**FIGURE 7:**
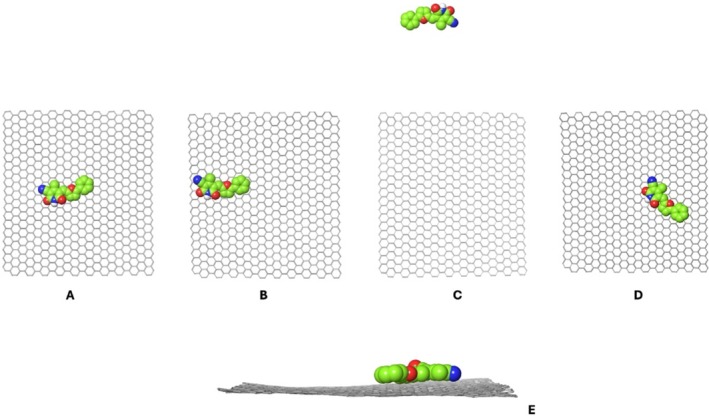
Representative snapshots of the MD simulation of **8** interacting with the graphene surface. Panels (A–D) show four key time points along the trajectory: (A) initial configuration (0 ns), (B) intermediate stage (150 ns), (C) intermediate stage (220 ns), and (D) final configuration (300 ns). Panel E shows a side view of compound **8** adsorbed flat on the graphene sheet. The ligand is displayed using a CPK representation, while the graphene sheet is shown in a hexagonal lattice representation. The images were generated using Maestro (Schrödinger LLC, New York, NY, United States).

MD simulations were performed to assess whether peptide functionalization of B60 affects drug loading. In particular, we investigated whether steric hindrance or electrostatic perturbations reduce the surface accessibility required for effective π–π interactions. To provide a comparative framework, the same computational protocol was applied to an analogous model system, **8**@(R11@B60), in which a single polyarginine chain was covalently anchored to the graphene sheet. This latter construct was intentionally designed as a simplified and schematic representation of the peptide‐functionalized surface, aimed at capturing the local influence of a cationic peptide moiety on the adsorption behavior of **8** without reproducing the full structural complexity of the experimental system. Such an approach allowed us to isolate the possible contribution of peptide‐induced steric and electrostatic effects on the loading process, while preserving the key interfacial features governing the interaction between compound **8** and the graphene surface. Figure 7 shows representative snapshots of the system at the beginning, during intermediate stages, and at the end of the MD production run, taken at 0, 150, 220, and 300 ns. As evident from the trajectory, although **8** moves away from the graphene plane in some frames, it does not definitively detach from the surface. Instead, it remains adsorbed and moves freely across the graphene plane throughout the simulation. Accordingly, the adsorbed state is not static; rather, the molecule undergoes continuous surface diffusion, sampling different positions and orientations while maintaining persistent contact with the graphene surface (Figure 7). This behavior indicates that adsorption is sufficiently stable to prevent desorption, yet sufficiently dynamic to allow progressive interfacial reorganization toward more favorable binding arrangements.

**FIGURE 8:**
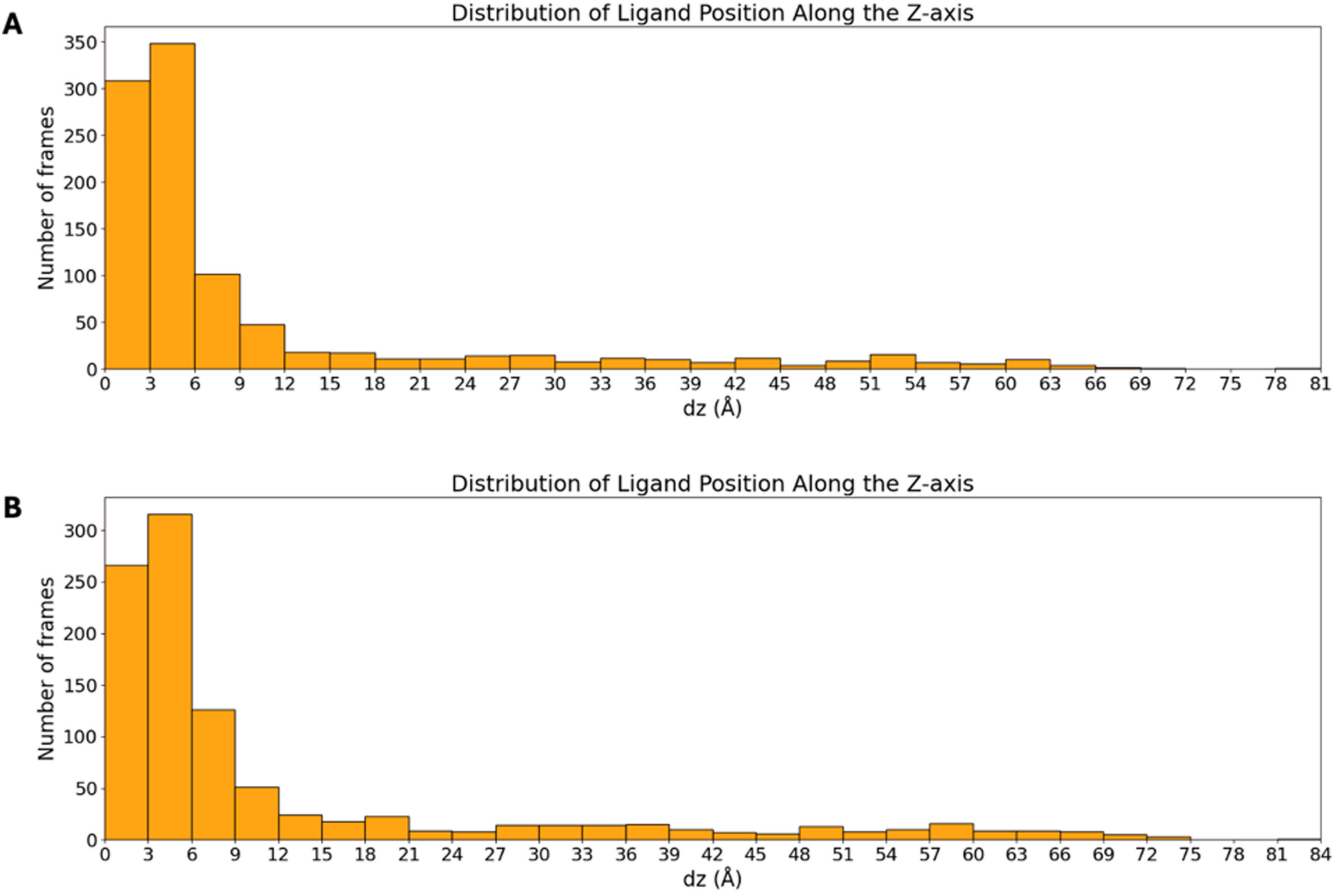
Distribution of the vertical distance (dz) separating the geometric center of compound **8** from the pristine graphene surface (A) and from the R11‐functionalized graphene surface (B) during the MD simulation. The histogram reports the number of frames falling within each distance interval (Å).

By the end of the trajectory, the complex remains firmly associated with the surface, stabilized by an extended contact area and a favorable arrangement of the aromatic moieties, with molecule‐graphene separations consistent with π‐π stacking interactions (typically ~3.3–3.6 Å) (Figure 7D). Consistently, the distribution of the ligand position along the z axis relative to the graphene surface indicates persistent localization within this interaction range throughout the trajectory (Figure 8A).

A pronounced population is observed at short interfacial separations, indicating that compound **8** predominantly remains adsorbed on the graphene surface. Several low‐population clusters are observed at larger distances, consistent with rare desorption events in which the molecule transiently moves away from the surface. Overall, the distribution indicates that compound **8** remains preferentially localized near the graphene surface, with only occasional excursions to larger separations. The adsorption of compound **8** on graphene is clearly demonstrated by the radial distribution function (RDF) profile.

**FIGURE 9:**
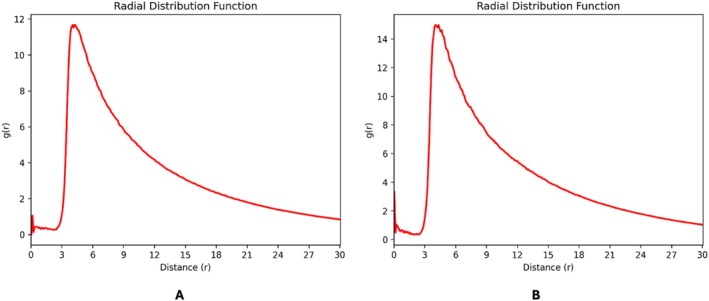
Radial distribution function (RDF), g(r), describing the spatial distribution of compound **8** relative to the pristine graphene surface (A) and the R11‐functionalized graphene surface (B) throughout the MD simulation. Plotted as a function of the radial distance, r (Å), g(r) represents the probability of finding the ligand at a given distance compared with a random distribution.

The RDF, g(r), as a function of the distance from the graphene surface, displays a sharp increase beginning at approximately 3 Å, followed by a pronounced first peak centered at about 4–5 Å (Figure 9A). This behavior indicates a highly populated preferred distance, consistent with adsorption of the ligand onto the graphene surface. Beyond this peak, g(r) gradually decreases with increasing distance and does not show additional well‐defined maxima, suggesting the absence of other preferred ligand–surface separations. At larger distances, g(r) approaches values close to unity, reflecting a progressive loss of spatial correlation and a more homogeneous distribution. Overall, the RDF supports a stable and well‐defined adsorption mode of compound **8** on graphene, with a marked preference for close contact with the surface.

**FIGURE 10:**
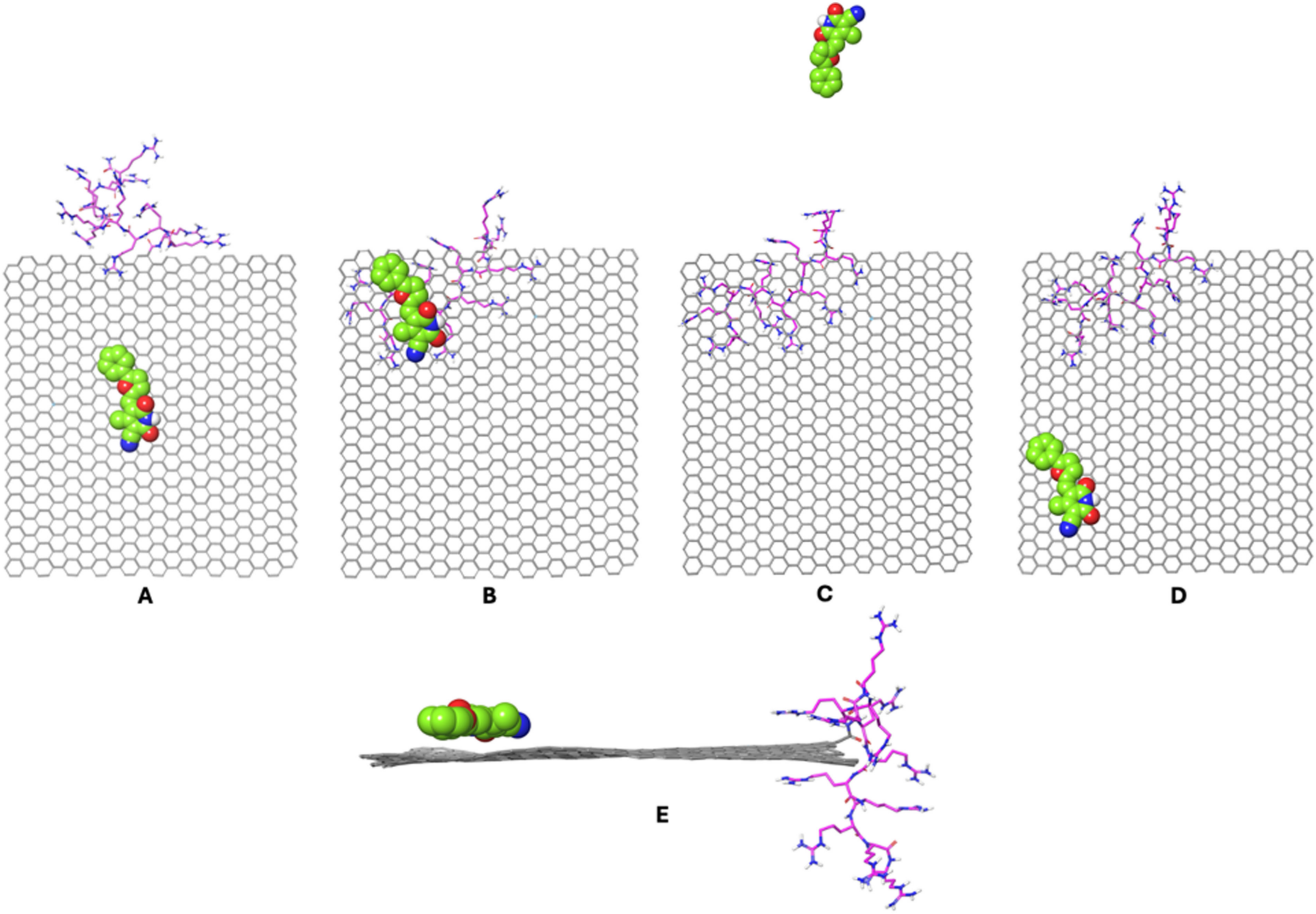
Representative snapshots of the MD simulation of **8** interacting with the graphene surface functionalized with R11. Panels (A–D) correspond to different time points along the trajectory: (A) initial configuration (0 ns), (B) intermediate state (150 ns), (C) intermediate state (235 ns), and (D) final configuration (300 ns). Panel E highlights the orientation of the polyarginine chain with respect to the graphene plane. Compound **8** is shown in CPK representation, R11 is displayed in thick tube representation, and the graphene surface is depicted as a hexagonal carbon lattice. The images were generated using Maestro (Schrödinger LLC, New York, NY, United States).

Notably, a closely analogous behavior was observed for the **8**@(R11@B60) model, where the covalently attached polyarginine chain was included as a schematic representation of the peptide‐functionalized surface. Although the cationic peptide fragment introduces a local steric and electrostatic perturbation, it does not suppress the ability of compound **8** to remain adsorbed while diffusing laterally across the graphene plane, nor does it hinder its eventual accommodation into a stable interfacial binding arrangement (Figure 10).

Throughout the trajectory, **8** preserves sustained contact with the graphene platform, although a greater propensity to explore configurations at increased distance from the surface is observed in the peptide‐functionalized model (Figure 8B). This feature likely reflects the local steric and electrostatic perturbation introduced by the polyarginine chain, which broadens the range of ligand positions without, however, promoting stable desorption. Indeed, despite the pronounced conformational fluctuations of the peptide moiety throughout the dynamics, the simulations consistently show that these do not hinder the adsorption process driven by favorable interactions with the graphene surface, as evidenced by the profiles of the vertical distance distribution (dz) and the RDF for the functionalized system (Figures 8B and 9B, respectively).

A quantitative comparison of the MD trajectories was also performed to assess the effect of R11 on compound **8** adsorption. As summarized in Table 1, the adsorption‐related descriptors obtained for **8**@B60 and **8**@(R11@B60) are highly comparable, supporting the conclusion that the peptide exerts only a limited influence on the overall adsorption process.

**TABLE 1:** MD‐derived descriptors characterizing the adsorption of compound **8** on graphene (**8**@B60) and on R11‐functionalized graphene (**8**@(R11@B60))^a^.

MD‐derived descriptors	8@B60	8@(R11@B60)
Mean adsorbate‐graphene distance (Å)	11.43 ± 15.61	12.11 ± 16.44
Most populated absorption distance range (Å)	3–4 Å	3–4 Å
Highest occupancy population	141/1003	148/1003
Number of adsorbate‐graphene frames (< 4 Å)	553/1003	499/1003
Ligand conformational stability (RMSD)	0.510 ± 0.15	0.537 ± 0.16
Residence time on graphene (%)	55.3%	49.75%

^a^ Distances were calculated as the shortest perpendicular distance between compound **8** and the mean plane of the graphene carbon atoms.

As shown in Table 1, the adsorption‐related descriptors obtained for the **8**@B60 and **8**@(R11@B60) systems are highly comparable. Both systems exhibit the same most populated adsorption distance range (3–4 Å) and similar average adsorbate‐graphene distances. Although a slight reduction in residence time and in the number of adsorbed frames is observed in the presence of R11, these differences are relatively small and indicate that the peptide does not substantially alter the adsorption behavior of compound **8**. Overall, the results suggest that adsorption remains primarily governed by interactions between compound **8** and the graphene surface.

**FIGURE 11:**
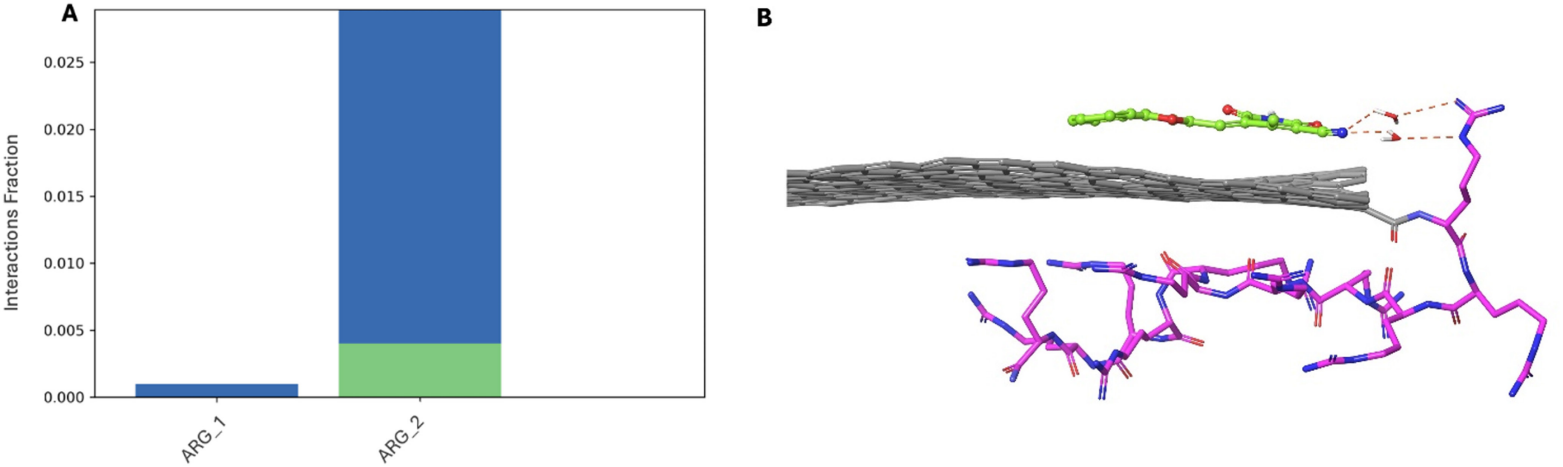
Fraction of interactions between compound **8** and the arginine residues of R11 during the MD simulation (A). The histogram shows, for each residue, the fraction of interaction occurrences along the trajectory, as determined using the Simulation Interaction Diagram tool available in Maestro (Schrödinger LLC, New York, NY, United States). In the color‐coded interaction scheme, blue represents water bridges, green hydrogen bonds. (B) Representative snapshot (272 ns) of the interactions between compound **8** and Arg1 of the polyarginine chain, mediated by two bridging water molecules. This configuration illustrates the transient water–bridge contact identified during the MD simulation.

To gain deeper insight into the nature of the interactions involving the peptide moiety, the interaction fractions between **8** and the arginine residues of R11 were analyzed throughout the MD trajectory (Figure 11). This analysis was undertaken to assess the contribution of individual arginine residues to ligand recognition and stabilization, and to determine whether peptide‐mediated contacts significantly influence the adsorption behavior of **8** in addition to its interaction with the graphene surface. The resulting interaction profile provides a residue‐resolved view of the peptide‐**8** interplay and offers further insight into the local intermolecular determinants governing the behavior of the system.

Overall, the observed interaction fractions are extremely low, indicating that contacts between compound **8** and the polyarginine chain are sporadic and are not maintained over any significant portion of the trajectory (Figure 11A). These contacts occur at variable positions along the peptide chain and are mediated predominantly by transient solvent‐assisted interactions rather than by a specific and persistent binding mode ruling out a well‐defined peptide‐mediated interaction pattern. Figure 11 shows the representative structure of the most populated cluster identified during the simulation, highlighting the dominant conformational state sampled by the system. A total of 16 clusters were obtained, with populations ranging from 1 to 30 frames. The most populated cluster, shown in Figure 11, represents the dominant binding mode of the system and reveals only transient, water‐mediated and nonspecific contacts between compound **8** and the flexible polyarginine segment. Such interactions arise from occasional spatial proximity and are consistently reproduced across independent MD replicas (see Supporting Information, Figures S5–S10). Taken together, these findings indicate that the peptide moiety contributes only marginally to ligand stabilization, whereas adsorption remains predominantly governed by favorable interactions with the graphene platform.

## 4 Conclusions

In this study, we demonstrated that the covalent functionalization with the CPP R11 can be effectively combined with π‐π‐driven loading in graphene‐based nanocarriers without compromising the intrinsic adsorption properties of the graphene surface. The orthogonal design strategy, where peptide grafting occurs selectively at the nanoparticle edges while the aromatic basal plane remains available for non‐covalent interactions, proves essential to preserve loading efficiency. Experimental results obtained with both the model compound PyCA and a protein kinase NEK6 inhibitor (compound **8**) clearly show that R11 functionalization does not significantly affect either loading capacity or thermally induced release. Spectroscopic and microscopic analyses confirm that the structural integrity of the graphene basal plane is maintained after peptide conjugation, while the peptide chains form a peripheral charged corona that does not interfere with π‐π interactions responsible for cargo binding. Consistently, MD simulations showed that compound **8** remains stably adsorbed on the graphene surface while retaining lateral mobility. These results indicate a dynamic yet persistent binding regime that is preserved even in the presence of the polyarginine moiety. Importantly, the stabilization of the π‐conjugated form of compound **8** upon adsorption highlights the active role of the graphene platform in modulating the physicochemical behavior of poorly water‐soluble drugs. The retention of high loading yields and controlled release profiles in the presence of the peptide further supports the robustness of this system. Overall, these findings provide clear evidence that edge‐selective peptide functionalization is fully compatible with basal‐plane adsorption in graphene nanocarriers. This architecture offers a promising platform for the development of peptide‐graphene hybrid systems that combine drug loading and controlled release of poorly soluble π‐conjugated bioactive molecules, such as NEK6 inhibitor compound **8**. Future studies will focus on the biological validation of the R11‐compound **8** platform in both oncological and neurodegenerative disease models. In particular, it will be important to determine whether the enhanced cellular uptake expected from the R11 moiety can be achieved without compromising the loading and release properties of the graphene carrier observed in the present study. More generally, the results indicate that peptide functionalization at the graphene edges can be combined with π‐π‐mediated loading of aromatic molecules without significant interference between the two processes. This finding may facilitate the development of related graphene‐based delivery systems incorporating alternative peptide sequences and biologically relevant π‐conjugated compounds.

## Funding

A.O. was supported by the Department of Chemical Sciences, University of Padova (P‐DiSC Project C93C22009260001 – MUR‐DE 2023 Chemical Complexity (C2)).

## Supporting information




**Figure S1:** UV–vis spectra (panel a) and Fluorescence spectra (panel b) of an aqueous dispersion of PyCA@(R11@B60) conjugates recorded before heating and after successive heating steps.
**Figure S2:** UV–vis spectra of aqueous dispersions of **8**@(R11@B60)_nW conjugates recorded after successive washing cycles (nW, *n* = 0–4).
**Figure S3:** UV–vis spectra of the supernatant obtained after centrifugation of the methanol–water dispersion of **8**@(R11@B60) and of a reference methanol–water solution of compound **8** after 24 h of sonication.
**Figure S4:** Thermal release after heating of the water dispersion of **8**@(R11@B60) at 70°C. UV–vis spectrum of the supernatant obtained after centrifugation of the water dispersion (green line) and of the same solution after addition of acetic acid (yellow line).
**Figures S5‐S7:** report the results obtained from independent replica 1 of the molecular dynamics (MD) simulations.
**Figures S8‐S10:** report the results obtained from independent replica 2 of the molecular dynamics (MD) simulations.

## Data Availability

The data that supports the findings of this study are available in the supplementary material of this article.
