# Supplementary material for: Edge‐Grafted Polyarginine Functionalization of Graphene Nanocarriers Maintains Noncovalent Aromatic Drug Loading
Source: J Pept Sci. 2026 Jul 5;32(8):e70114. doi: 10.1002/psc.70114 (PMC13333320; doi:10.1002/psc.70114)
Supplement: Supplementary file 1 — Figure S1: UV–vis spectra (panel a) and Fluorescence spectra (panel b) of an aqueous dispersion of PyCA@(R11@B60) conjugates recorded before heating and after successive heating steps. Figure S2: UV–vis spectra of aqueous dispersions of 8@(R11@B60)_nW conjugates recorded after successive washing cycles (nW, n = 0–4). Figure S3: UV–vis spectra of the supernatant obtained after centrifugation of the methanol–water dispersion of 8@(R11@B60) and of a reference methanol–water solution of compound 8 after 24 h of sonication. Figure S4: Thermal release after heating of the water dispersion of 8@(R11@B60) at 70°C. UV–vis spectrum of the supernatant obtained after centrifugation of the water dispersion (green line) and of the same solution after addition of acetic acid (yellow line). Figures S5‐S7: report the results obtained from independent replica 1 of the molecular dynamics (MD) simulations. Figures S8‐S10: report the results obtained from independent replica 2 of the molecular dynamics (MD) simulations. [file PSC-32-e70114-s001.docx]

SUPPORTING INFORMATION FOR

**Edge-Grafted Polyarginine Functionalization of Graphene Nanocarriers Maintains Noncovalent Aromatic Drug Loading**

Beatrice Scagnoli,^1,^**^#^** Alessandro Semeraro,^1,^**^#^** Kaiyue Hu,^2^ Alberto Ongaro,^3,§^ Agnese Pavan, ^3^ Luigi Brambilla,^2^ Chiara Castiglioni,^2^ Maria Cristina De Rosa,^1^ Giuseppe Pappalardo,^4^ Giuseppina Sabatino,^4,^* Michele Maggini.^3,5^

1. Istituto di Scienze e Tecnologie Chimiche Giulio Natta (SCITEC)-CNR, Roma, 00168 Roma, Italy;
2. Dipartimento di Chimica, Materiali e Ingegneria Chimica Giulio Natta, Politecnico di Milano, 20133 Milano, Italy;
3. Dipartimento di Scienze Chimiche, Università di Padova, 35131 Padova, Italy;
4. CNR-Istituto di Cristallografia, 95126 Catania, Italy;
5. Istituto di Chimica della Materia Condensata e di Tecnologie per l’Energia (ICMATE)-CNR, 35127 Padova, Italy.

(#) These authors contributed equally to this work.

(§) present address: Dipartimento di Scienze Chimiche, Farmaceutiche e Agrarie, Università di Ferrara, 44121 Ferrara, Italy

(*) Corresponding author: [giuseppina.sabatino@cnr.it](mailto:giuseppina.sabatino@cnr.it)

**TABLE OF CONTENT**

**Figure S1.** UV-vis spectra (panel a.) and Fluorescence spectra (panel b.) of an aqueous dispersion of PyCA@(R11@B60) conjugates recorded before heating and after successive heating steps

**Figure S2.** UV-vis spectra of aqueous dispersions of **8**@(R11@B60)_nW conjugates recorded after successive washing cycles (nW, n = 0-4).

**Quantitative determination of the loading yield in 8@(R11@B60) conjugates.**

**Figure S3.** UV-vis spectra of the supernatant obtained after centrifugation of the methanol-water dispersion of **8**@(R11@B60) and of a reference methanol-water solution of compound **8** after 24 h of sonication.

**Figure S4.** Thermal release after heating of the water dispersion of **8**@(R11@B60) at 70°C. UV-vis spectrum of the supernatant obtained after centrifugation of the water dispersion (green line) and of the same solution after addition of acetic acid (yellow line).

**Figures S5-S7** report the results obtained from independent replica 1 of the molecular dynamics (MD) simulations.

**Figures S8-S10** report the results obtained from independent replica 2 of the molecular dynamics (MD) simulations.

| 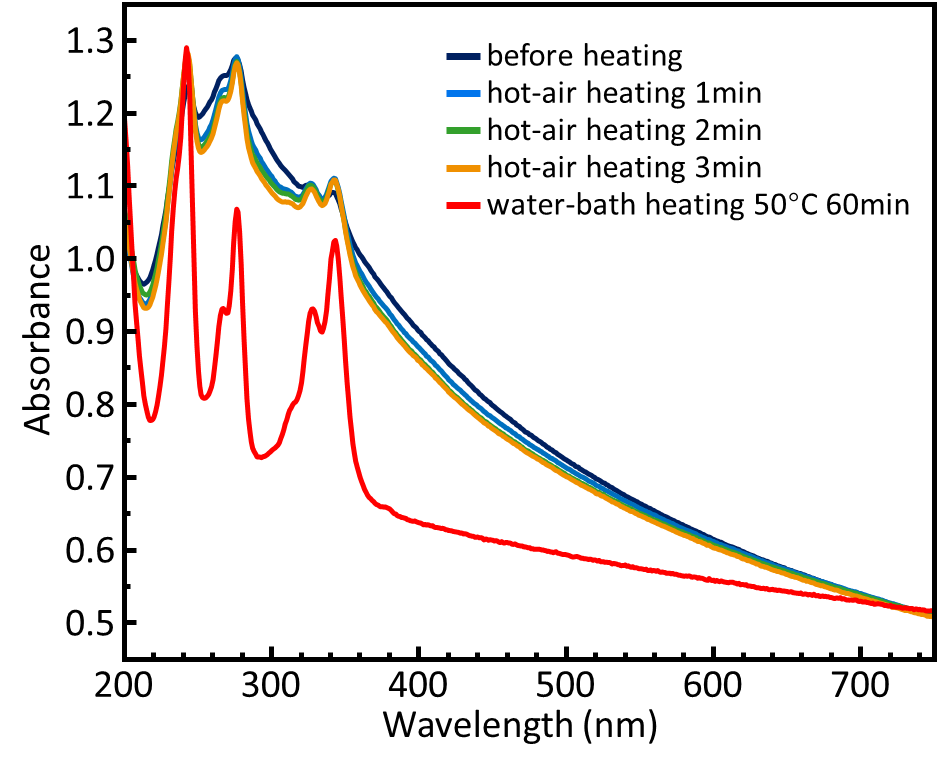 |
| --- |
| **a** |
| 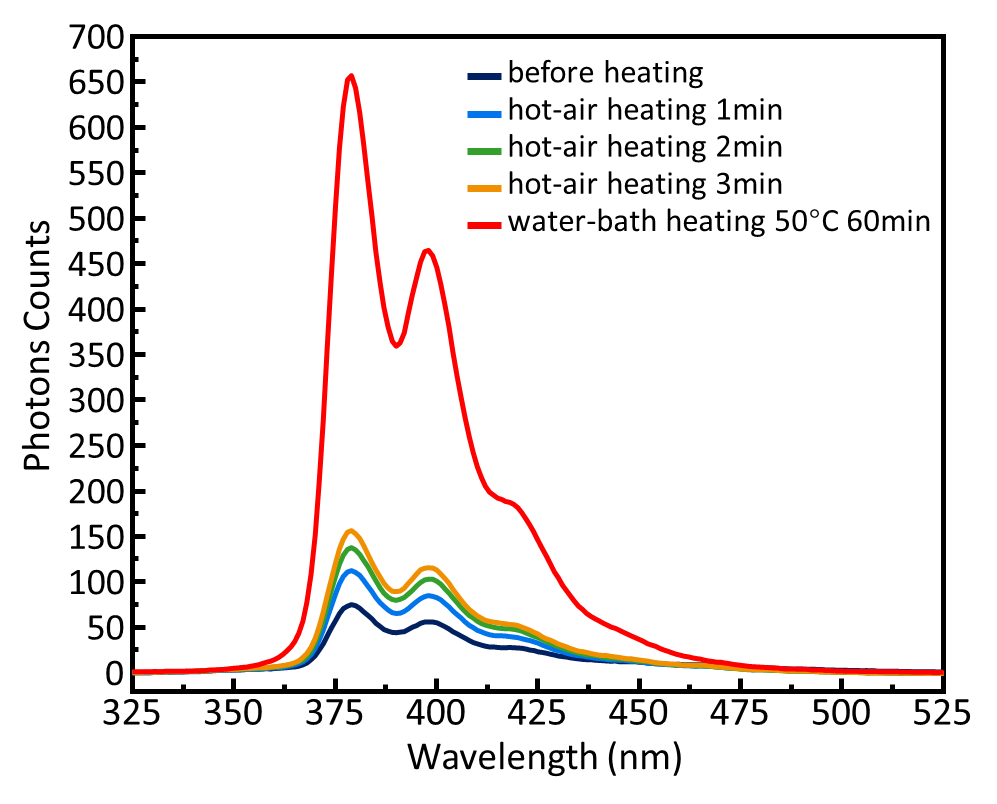 |
| **b** |

**Figure S1**. UV-vis spectra (panel **a**) and Fluorescence spectra (panel **b**) of an aqueous dispersion of PyCA@(R11@B60) conjugates recorded before heating and after successive heating steps, during which the sample was exposed to a hot air flux. The spectrum obtained after maintaining the sample at a constant temperature of 50 °C for 1 hour is also shown.

**
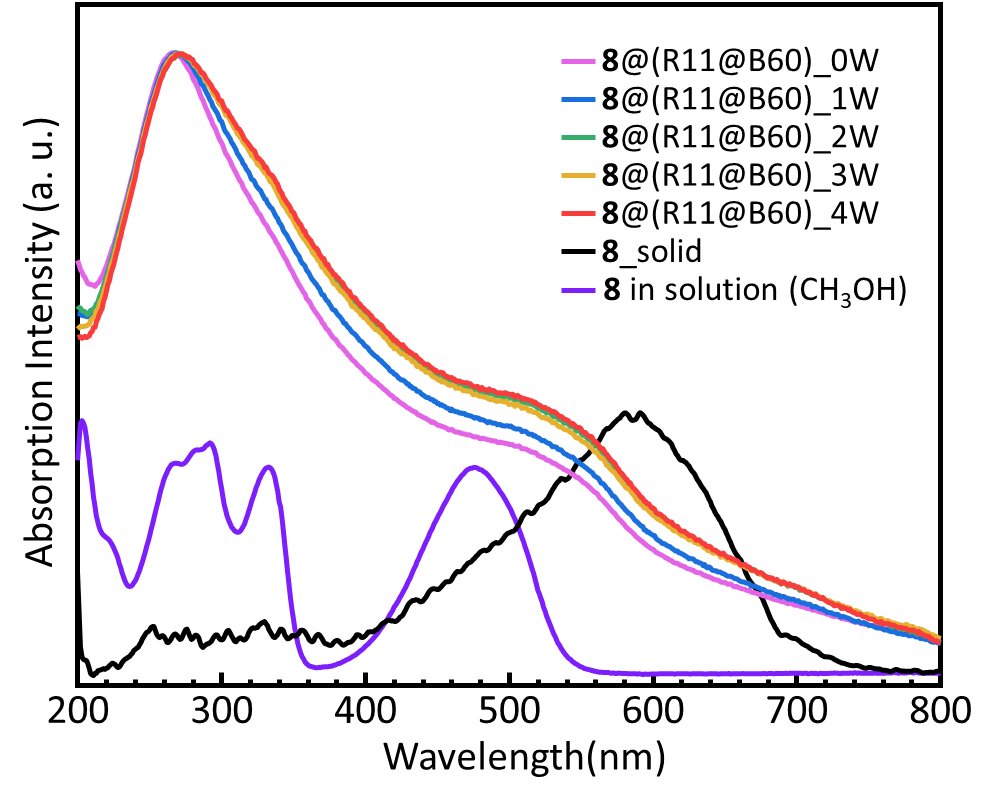
**

**Figure S2.** UV-vis spectra of aqueous dispersions of **8**@(R11@B60)_nW conjugates recorded after successive washing cycles (nW, n = 0-4). The spectra result from the superposition of the absorption bands of the graphene platform (R11@B60) and those of **8**, whose spectrum is also shown for comparison. A moderate decrease in the intensity of the **8** absorption bands is observed after the first washing step, whereas subsequent washing cycles produce nearly unchanged spectral profiles.

**Quantitative determination of the loading yield in 8@(R11@B60) conjugates.** To estimate the fraction of **8** loaded onto R11@B60, two complementary methods were developed based on the intensity of characteristic UV-vis absorption bands of compound **8**. In the first approach, the yield of the conjugation reaction was estimated assuming that nanoparticles loaded with **8** can be separated from the reaction mixture by centrifugation. Under these conditions, the molecules of **8** that are not bound to the platform remain in the supernatant (hereafter referred to as the *“first water”*). The concentration of free **8** in the “first water” was determined by UV-vis spectroscopy through comparison with a reference solution. Because compound **8** may undergo chemical changes during the prolonged sonication used in the preparation of the conjugate, the reference solution was prepared under identical conditions (CH₃OH 2 ml + H₂O 6.2 ml) and sonicated for 24 h. The resulting transparent solution shows the characteristic absorption band of the colorless Form I of **8** at 330 nm (Figure S3), with spectral features matching those observed for the “first water”. The ratio between the intensities of the 330 nm bands in the two spectra therefore provides a reliable estimate of the concentration of free **8**. This analysis indicates that the “first water” contains 8% of the initial amount of **8**, corresponding to a loading yield of 92% of compound 8 on the R11@B60 conjugate.


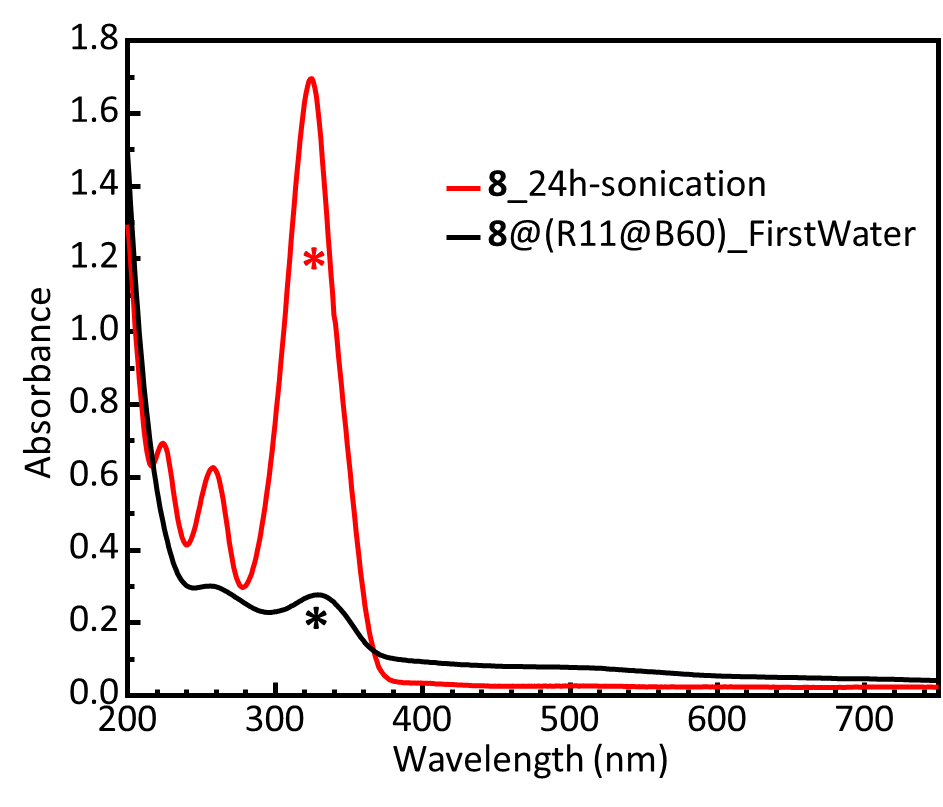


**Figure S3.** UV-vis spectra of the supernatant obtained after centrifugation of the methanol-water dispersion of **8**@(R11@B60) (black line) and of a reference methanol-water solution of compound **8** after 24 h of sonication (red line). Asterisks indicate the absorption peaks of **8** used for quantification. Both solutions were prepared from the same initial concentration of compound **8**.

The loading determination described above likely overestimates the amount of compound **8** present in the final sample, i.e., the aqueous dispersion of the conjugates. This dispersion undergoes four washing cycles with water to remove weakly bound or free **8** molecules as well as residual methanol. An alternative estimate can be obtained from the final aqueous dispersion after thermal release at 70 °C. After release, the R11@B60 nanoparticles are removed by centrifugation and the supernatant containing the released **8** is analyzed (Figure S4).

| **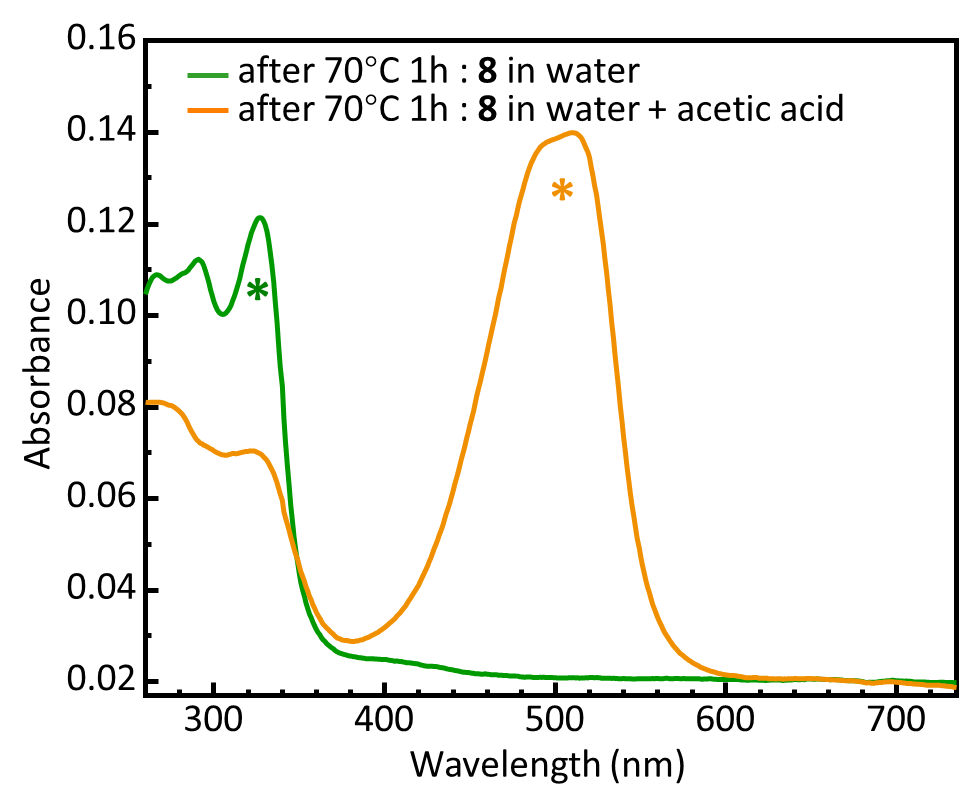** |
| --- |

**Figure S4.** Thermal release after heating of the water dispersion of **8**@(R11@B60) at 70°C. UV-vis spectrum of the supernatant obtained after centrifugation of the water dispersion (green line) and of the same solution after addition of acetic acid (orange line). Acidification promotes conversion of compound **8** from the colourless Form I to the coloured Form II. Asterisks indicate the absorption peaks of **8** used for quantification.

The spectrum shows the characteristic absorption profile of the colorless Form I of compound **8**. Although **8** is initially loaded onto the platform in the orange Form II, its conversion to the colorless Form I after release in water is consistent with the spontaneous hydration reaction previously reported [1]. The released molecules were quantified from the absorption band at 330 nm using as reference a solution of pure **8** in H₂O-CH₃OH allowed to undergo complete discoloration. This analysis indicates that 68% of the initial amount of **8** used for conjugate preparation is present in solution after release. Assuming quantitative release, this value reflects the overall efficiency of the preparation process leading to the stable aqueous dispersion of **8**@(R11@B60). The ~30% decrease relative to the previous estimate is reasonable, as some drug may be lost during washing and the release process may not be fully quantitative. A lower estimate was obtained from the same solution using a complementary UV-vis measurement after addition of a few drops of acetic acid, which promotes conversion of **8** from the colorless Form I to the orange Form II. In this case, quantification is based on the characteristic absorption of Form II at 480 nm, giving a loading of 46%. This value is likely underestimated, since complete conversion to Form II cannot be achieved and the reverse reaction to the colorless form occurs over time even under acidic conditions. Although these estimates should be regarded as semi-quantitative, they consistently indicate efficient conjugation of **8** even in the presence of R11 grafted onto the B60 GNP. Applying the same procedures to **8**@B60 gives comparable values: 75% from the UV-vis spectrum of the “first water”, and 73% or 50% from the UV-vis spectrum after thermal release of the drug (absorption at 330 nm (Form I) and 480 nm (Form II after acid-catalised dehydration), respectively).


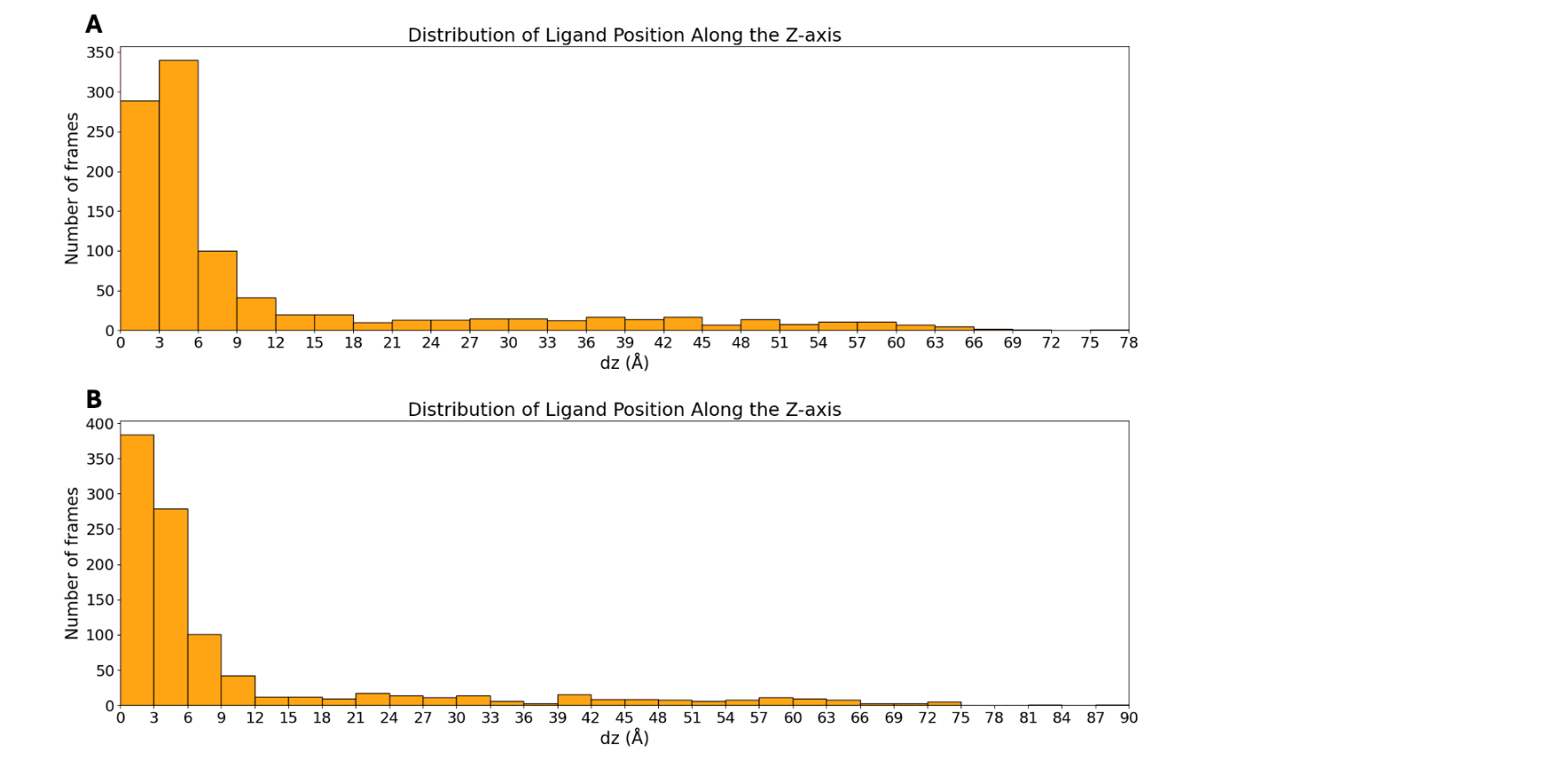


**Figure S5.** Distribution of the vertical distance (dz) separating the geometric center of compound **8** from the pristine graphene surface (A) and from the R11-functionalized graphene surface (B) from MD replica 1. The histogram reports the number of frames falling within each distance interval (Å).


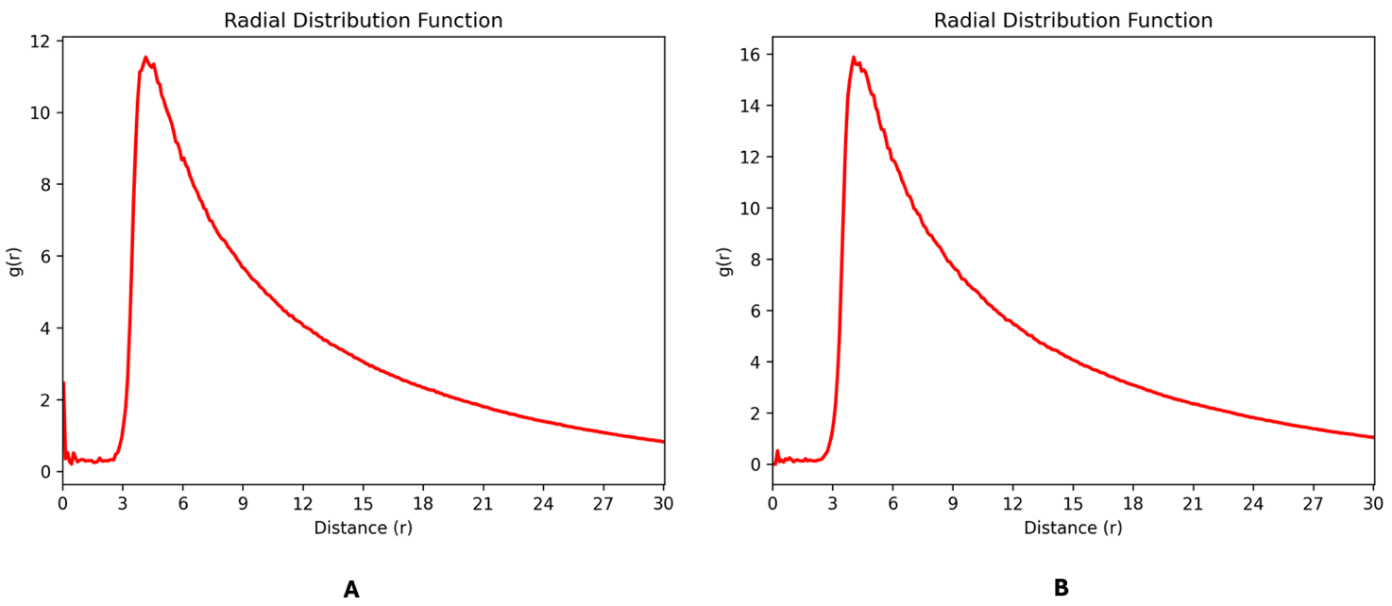


**Figure S6.** Radial distribution function (RDF), g(r), describing the spatial distribution of compound **8** relative to the pristine graphene surface (A) and the R11-functionalized graphene surface (B) from MD replica 1. Plotted as a function of the radial distance, r (Å), g(r) represents the probability of finding the ligand at a given distance compared with a random distribution


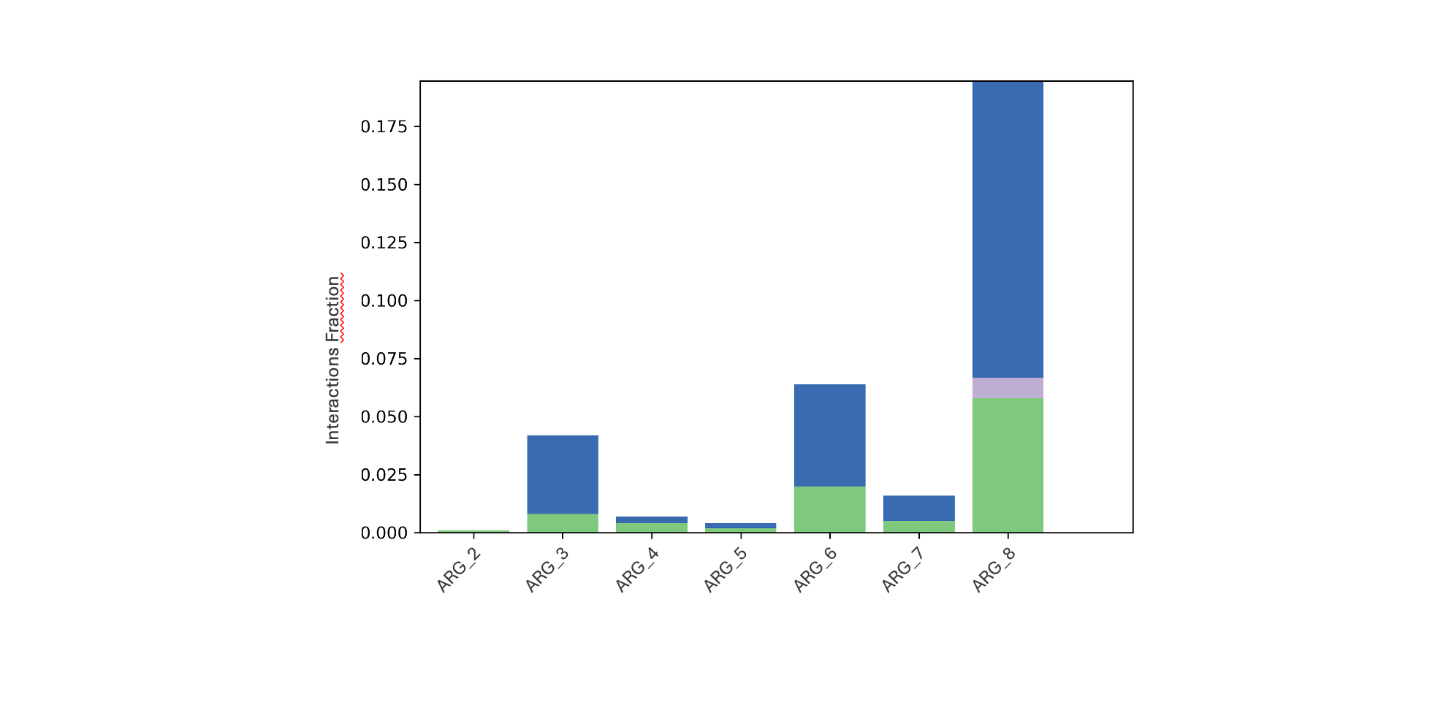
**Figure S7.** Fraction of interactions between compound **8** and the arginine residues of R11 from MD replica 1. The histogram shows, for each residue, the fraction of interaction occurrences along the trajectory, as determined using the Simulation Interaction Diagram tool available in Maestro (Schrödinger, LLC, New York, NY, USA). In the color-coded interaction scheme, blue represents water bridges, green represents hydrogen bonds and grey represents hydrophobic contacts.


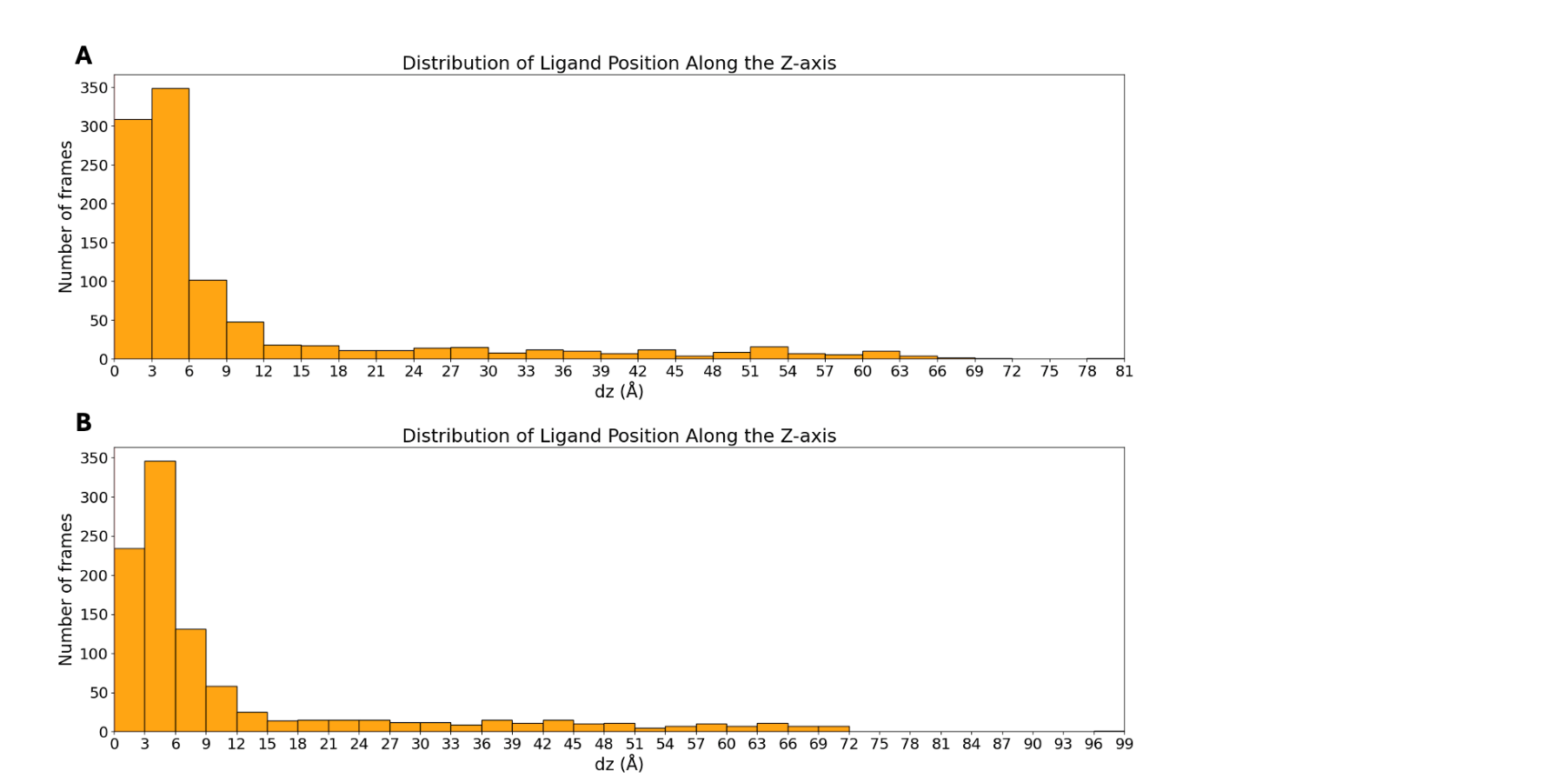


**Figure S8.** Distribution of the vertical distance (dz) separating the geometric center of compound **8** from the pristine graphene surface (A) and from the R11-functionalized graphene surface (B) from MD replica 2. The histogram reports the number of frames falling within each distance interval (Å).


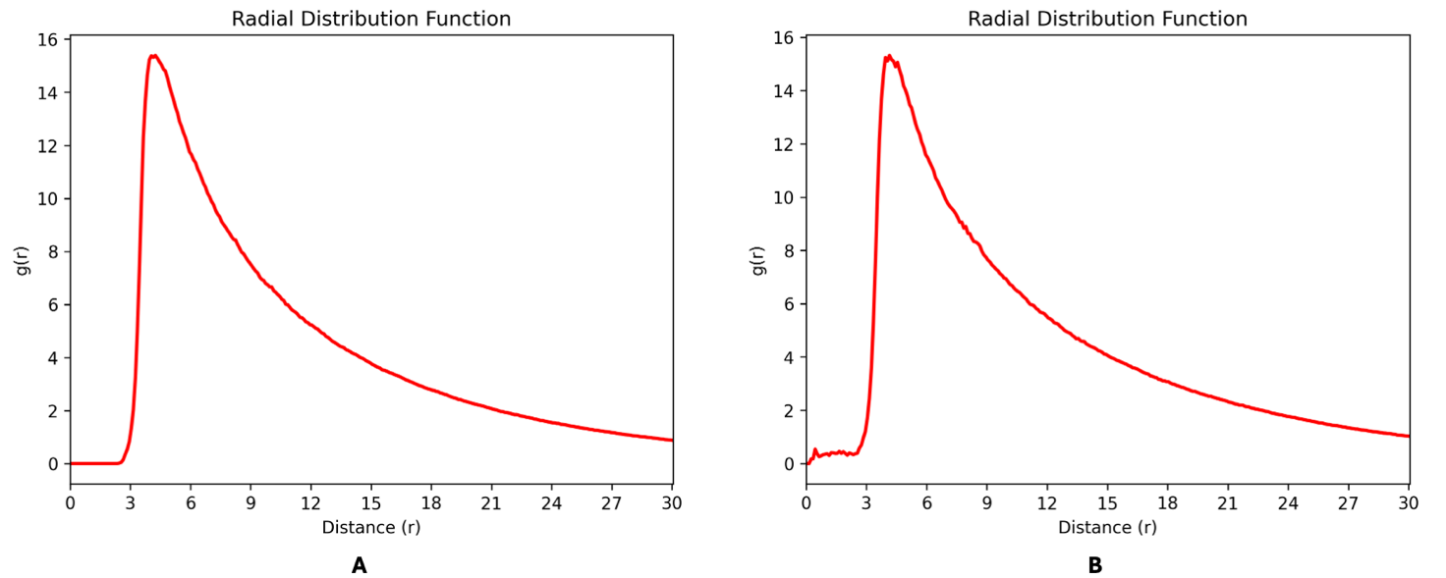


**Figure S9.** Radial distribution function (RDF), g(r), describing the spatial distribution of compound **8** relative to the pristine graphene surface (A) and the R11-functionalized graphene surface (B) from MD replica 2. Plotted as a function of the radial distance, r (Å), g(r) represents the probability of finding the ligand at a given distance compared with a random distribution.

**
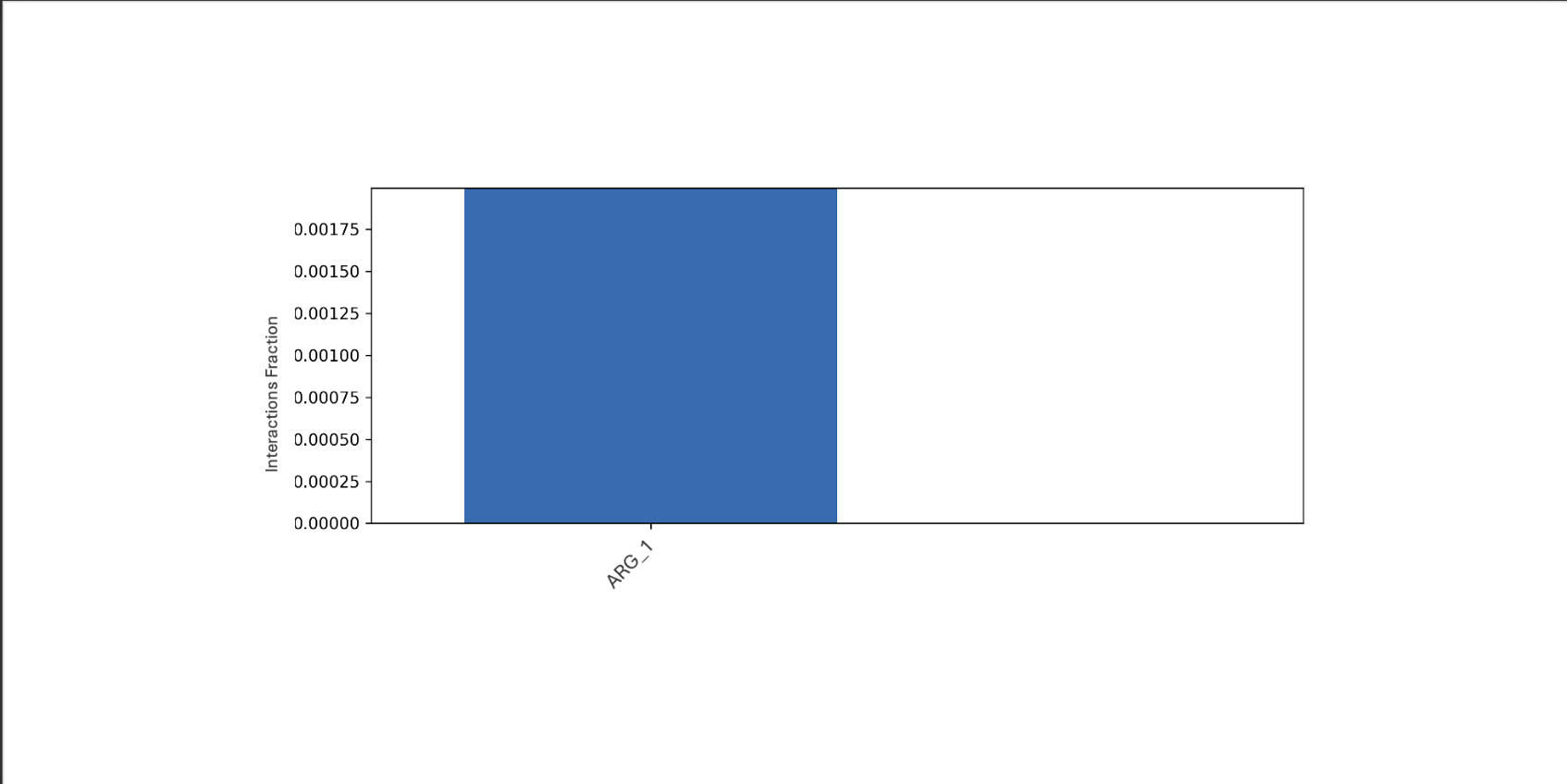
**

**Figure S10.** Fraction of interactions between compound **8** and the arginine residues of R11 from MD replica 2. The histogram shows, for each residue, the fraction of interaction occurrences along the trajectory, as determined using the Simulation Interaction Diagram tool available in Maestro (Schrödinger, LLC, New York, NY, USA). In the color-coded interaction scheme, blue represents water bridges.

**Bibliography**

[1] Hu K, Pavan A, Semeraro A, Ongaro A, Brambilla L, De Rosa MC, Tommasini M, Castiglioni C, Maggini M, Drug Loading and Release: Development and Characterization of a Novel Therapeutic Agent-Nanographene Conjugate. J. Phys. Chem. B. 2025; 129 (36): 9097-9112
